# Glycine Betaine-Functionalized Ionic Liquids: Design of Versatile Bioplatform for Incorporating Biologically Active Moieties into Cations

**DOI:** 10.1021/acsmedchemlett.5c00280

**Published:** 2025-08-19

**Authors:** Witold Stachowiak, Marcin Wysocki, Lorenzo Guazzelli, Michał Niemczak

**Affiliations:** † Faculty of Chemical Technology, 49632 Poznan University of Technology, Poznan 60-965, Poland; ‡ Chair and Department of Inorganic and Analytical Chemistry, 37807 Poznan University of Medical Sciences, Poznan 60-806, Poland; § Department of Pharmacy, 9310 University of Pisa, 56126 Pisa, Italy

**Keywords:** Glycine Betaine, Functionalized Ionic Liquids, Biologically Active, Design of Versatile Bioplatform, Cations

## Abstract

We report a novel series of ILs formed by coupling glycine betaine with the biologically active herbicide MCPA via an ester bond. ILs were synthesized through a 2-step *O*-alkylation process, followed by product isolation using flash chromatography. The alkyl chain length between the carboxylic groups determines solubility and octanol–water partition coefficient, whereas the rate of their hydrolysis is highly influenced by the pH of aqueous solution. All compounds retained the biological activity of the incorporated herbicide.

## Graphical abstract

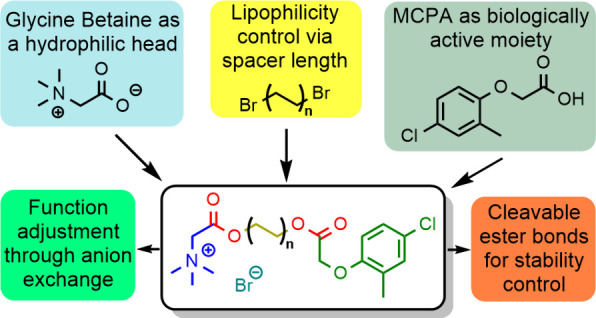

Ionic liquids (ILs) have garnered significant attention over the past three decades due to their unique properties, making them attractive chemicals for green chemistry, drug delivery, and agrochemical formulations.^1–3^ A common strategy in designing API-ILs (active pharmaceutical ingredient – ionic liquids) involves pairing pharmaceutically active anions with either synthetic or naturally derived cations.^1,3,4^ Notable examples include the combination of APIs in anionic form, such as ibuprofenate, salicylate, and theophyllinate, with cations like choline, benzalkonium, or alkylimidazolium.^5–7^ More recently, this concept has been extended to agrochemicals, where herbicidal ionic liquids (HILs) were formed by pairing herbicide anions, such as phenoxy acids or sulfonylureas, with diverse quaternary ammonium cations.^2^ It has been also noticed that linking two biologically active ions together gives access to diverse bifunctional ILs, such as lidocainium salicylate, benzalkonium ibuprofenate, etc.^6,7^ Noteworthy, some of the aforementioned anionic biologically active substances can be also introduced into the cation of ILs. Choline-based ILs, for instance, have been successfully employed in the synthesis of functionalized herbicide esters of various herbicides,^8,9^ where the herbicidally active moiety was incorporated into the cation via an ester linkage. ILs containing an ester bond within the cation are commonly referred to in the literature as ’esterquats’ to emphasize their key advantages, such as improved bioavailability and enhanced environmental safety, which arise from the presence of a cleavable bond.^10,11^ Moreover, synthesis of choline-based esterquats by proline-mediated Knoevenagel–Doebner condensation^12^ led to new forms of sinapine with various potential properties (anti-UV, antioxidant, anti-inflammatory, anticancer, and/or antimicrobial properties). This architectural option allows for controlling the release of the active substance from the cation and calls for synergistic effects by pairing such cations with functional anions, e.g., API or herbicide. Despite the growing interest in functionalized ILs, glycine betaine (GB), a naturally occurring zwitterionic compound, remains underexplored as a platform for such modifications even though it has been utilized in other green chemistry applications.^10,13–16^ Hence, unlike choline esters, there are no prior reports of ester-linked biologically active agents incorporated into GB. This analogue of natural amino acids offers significant advantages over other cationic platforms, such as choline, including its biocompatibility, truly natural origin, lack of toxicity, and low production costs. Importantly, unlike traditional synthetic strategies for esterification relying on toxic reagents, like thionyl chloride, the esterification process using GB can be accomplished without the addition of highly hazardous chemicals, further enhancing its utility in the environmentally friendly synthesis. A comprehensive comparison between our MCPA-based esterquats and other HILs reported in the literature, highlighting differences in synthetic strategies and application-related advantages, such as those summarized by Wilms et al.,^2^ is provided in the Supporting Information (Table S1).

First, we report a strategy for accessing novel alkylating agents containing an alkyl spacer ranging from butyl to dodecyl (**1**-**5**), obtained through the functionalization of 4-chloro-2-methylphenoxyacetic acid (MCPA herbicide), a widely used selective herbicide for protecting cereals against dicotyledonous weeds such as lambsquarters, cornflower, and white mustard. The most challenging aspect of their synthesis was the development of a purification procedure, which required the use of flash chromatography on silica gel, followed by its subsequent optimization. In the second step, the purified bromo esters of MCPA herbicide were used for the *O*-alkylation of GB, yielding biologically active esterquats with hydrolyzable ester bonds (**6**-**10**). It should be emphasized that, to date, only one method has been reported in the literature for obtaining choline-based derivatives functionalized via the incorporation of a biologically active moiety. This method involves the esterification of 2-dimethylaminoethanol with acid chlorides, followed by quaternization with alkyl bromides.^8,9^ However, these derivatives exhibit significant drawbacks compared with GB-based esterquats. Although the choline cation occurs naturally, its synthetic production is complex, and its chemical modification toward esterquats requires highly toxic reagents, such as thionyl chloride.^8,9,17^ Moreover, the absence of a spacer in the resulting products limits the tunability of their physicochemical properties. Therefore, the concept based on functionalization of GB instead of choline brings a lot of specific benefits that have been summarized in Figure 1.

**Figure 1:**
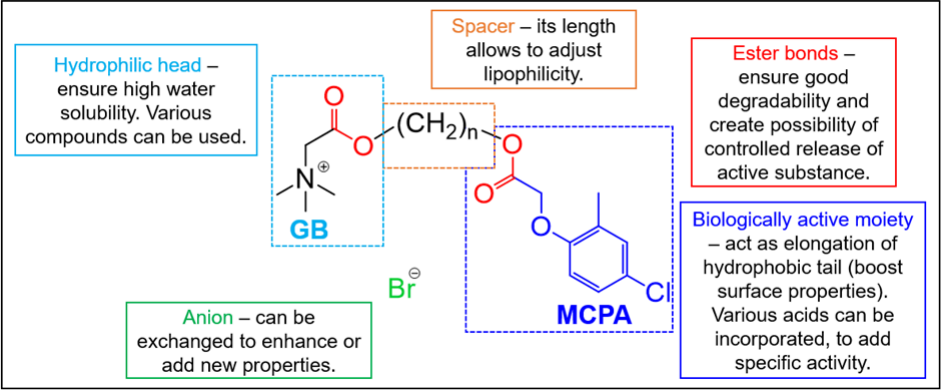
Concept of functionalized esterquats obtained from glycine betaine.

The elaborated two-step method of synthesis of functionalized ILs incorporating GB and MCPA is shown in Scheme 1. In the first step, the potassium salt of selected active ingredient (MCPA) was esterified with 1,ω-dibroalkane in ACN:MeOH v:v 1:1 solution or dimethylformamide to obtain ω-bromoalkyl 4-chloro-2-methylphenoxyacetates. Due to the fact that MCPA potassium salt is insoluble in acetonitrile, the addition of methanol allowed the substrate to solubilize and substantially faciliated the first *O*-alkylation. Other solvent systems (ACN:MeOH v:v 3:1, ACN:MeOH v:v 1:3, MeOH, and dimethyl sulfoxide) were tested and assessed as unsuitable for this process (more information in pp S4–S8) due to formation of byproducts. After the reaction, the solvents were evaporated, and functionalized alkylating agents (**1**-**5**), demonstrated in Scheme 1, were separated by vacuum distillation (for n = 4 or 6) or by flash column chromatography on silica gel 60 with hexane:ethyl acetate as eluent (for n = 8–12). This step was crucial due to formation of various side products with similar properties that hindered separation of the desired compounds with the use of other techniques (details are in pp S4–S8 and Tables S2–S3). Subsequently, the purified semiproducts (**1**-**5**) were used for *O*-alkylation of GB (Scheme 1) in acetonitrile to obtain functionalized GB and MCPA esterquats (**6**-**10**). Ensuring thorough mixing was essential, as betaine dissolves during the reaction, and insufficient agitation was found to significantly prolong the reaction time.^18^ The detailed methodology as well as the established structures of isolated side products are provided in the Supporting Information (see pp S4–S8 and Figure S1). The structures of all designed products and major side products were determined and confirmed using FT-IR (Fourier-transform infrared), ^1^H and ^13^C NMR (nuclear magnetic resonance), as well as MS (mass spectroscopy) techniques (Figures S2–S50). Analysis of the collected data, particularly NMR spectra, proved that the developed methodology enabled the generation of pure functionalized esterquats. The exemplified comparison between substrates, semiproduct (**3**), and the final product (**8**) containing 8-carbon atoms in a spacer is shown in Figure 2. The signals from aromatic protons between 6.86 and 7.22 ppm, characteristic for the MCPA aromatic ring, are also present in the spectrum of **8**. The additional singlet at 4.72 ppm was attributed to the methylene protons in the α-position relative to the carbonyl group. The signals in the range of approximately 1.00 to 2.00 ppm support the presence of an alkyl spacer in the product. Noteworthy, protons from methylenes adjacent to the bromide or oxygen atoms occurred as triplets at higher values of chemical shifts: at 3.51 and 4.10 ppm, respectively. In consequence of a second *O*-alkylation of GB, the signal at 3.51 ppm disappeared, and a new triplet appeared at 4.18 ppm, indicating formation of the second ester bond between the compound semiproduct (3) and GB. Additionally, a singlet at 3.30 ppm from three methyl groups, along with a singlet at 4.60 ppm from the GB methylene group, confirm the successful coupling of GB and MCPA within the esterquat strategy.

**Scheme 1:**
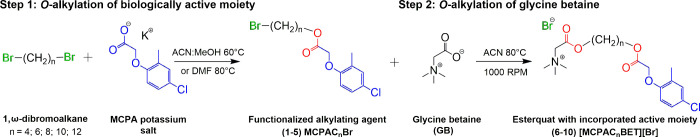
Synthesis of Functionalized Alkylating Agents of MCPA Herbicide (**1**-**5**) and Esterquats of Glycine Betaine and MCPA (**6**-**10**)

**Figure 2:**
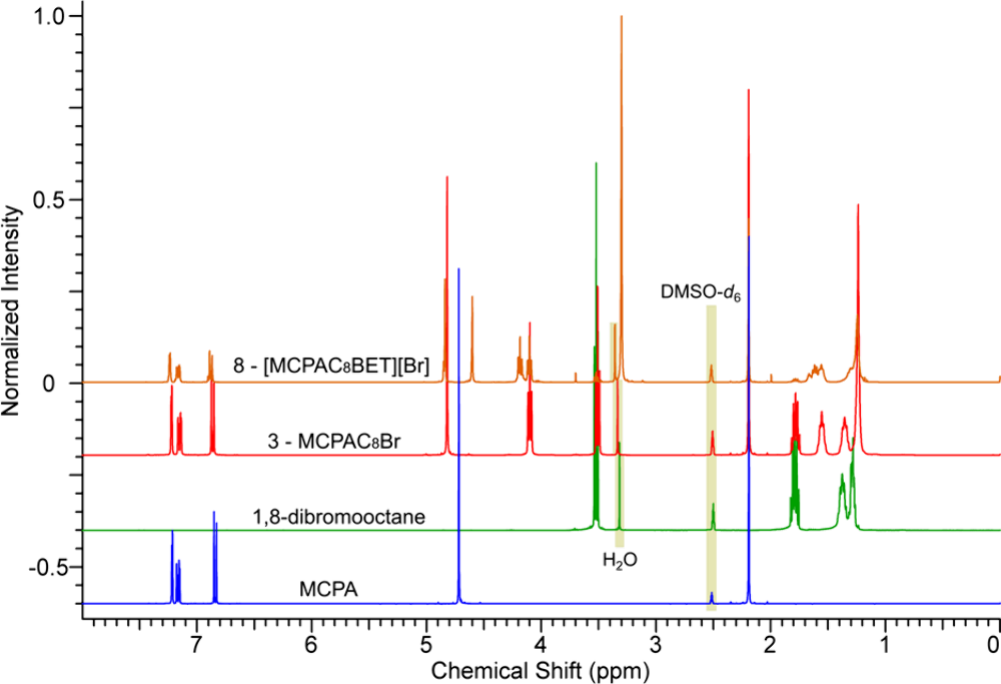
Comparison of ^1^H NMR spectra of substrates (MCPA – blue, 1,8-dibromooctane – green), semi-product (**3** – red), and esterquat (**8** – orange).

Table 1 lists all the obtained semiproducts (**1**-**5**) and esterquats (**6**-**10**) along with their yields, water content, melting points, and calculated logarithm of the K_OW_ coefficient. Due to side reactions, the functionalized alkylating agents were obtained with moderate yields (40–55%). Therefore, future optimization should aim to minimize byproduct formation to less than 10–20%. An interesting alternative to dibromoalkanes worth investigating is the use of chlorobromo- or chloroiodoalkanes, as this approach may reduce side reactions and simplify the purification process. However, their significantly higher cost greatly limits the practical applicability of this strategy. Nonetheless, the second step, which was much more straightforward to optimize, consistently resulted in excellent yields (>97%). The alkylating agents (**2**-**5**) were established as white solids with low melting points (below 50 °C), except for compound **1**, which was a colorless liquid. Interestingly, linking of **1**-**5** with GB introduces also an ionic bond into the final products, which leads to a significant increase in the melting points, reaching up to 100 °C for compound **8**. However, further elongation of the spacer causes a decrease in the melting point to 81 and 73 °C for **9** and **10**, respectively. This is mainly due to the weakening of electrostatic interactions, enhanced van der Waals forces, and disrupted crystal packing, which collectively reduce the lattice energy and hinder crystallization of many ILs.^19^ Nonetheless, it should be noted that among the obtained salts, only **8** does not melt below 100 °C; hence, the rest of the compounds (**6**, **7**, **9**, **10**) fulfill the requirements to be classified as ILs.^20^ Interestingly, compound **6** exhibits polymorphism, as evidenced by two distinct melting point events: the first at 77 °C and the second after prolonged storage at 125 °C. The water content in compounds **1**-**5** occurred in the range from 0.2 to 0.8%, while for esterquats **6**-**10** the measured values were considerably higher (0.9–2.7%). These data are consistent with our expectations due to the increased hydrophilicity of final products caused by the introduction of the highly hydrophilic GB and the ionic bond.

**Table 1:** Synthesized Compounds

No	n	Yield (%)	MP^a^ (°C)	H_2_O^b^ (%)	Log K_OW_
Alkylating agents - MCPAC_n_Br					
1	4	40–55^c^	—	0.22	4.6
2	6	44–50^c^	35.2–37.7	0.26	5.6
3	8	45–50^c^	40.0–42.5	0.34	6.6
4	10	46	42.0–45.0	0.83	7.6
5	12	50	41.8–43.2	0.55	8.6
Esterquats - [MCPAC_n_BET][Br]					
6	4	99	76.5–79.5;	1.60	0.1
			125–130^d^		
7	6	98	98.0–106.0	0.98	1.0
8	8	99	100.0–105.0	0.89	2.0
9	10	97	80.6–84.8	2.67	3.0
10	12	97	73.0–77.0	2.12	4.0

a MP - melting point.

b H_2_O - water content.

c Range established on a basis of 3 independent experiments.

d Two distinctive melting points were recorded.

In accordance with Ghose filter, calculated log K_OW_ of active ingredients should be in a range between −0.4 and 5.6 for their good bioavailability, which is also valid in the case of herbicides.^21^ However, the ideal liphophilicity highly depends on the type of action of the active substance, the route of its administration (application), and its final destination in an organism.^22,23^ Thus, log K_OW_ affects many aspects regarding the performance of applied herbicide or API, such as solubility, absorption, membrane permeability, distribution, and metabolism.

Substances characterized by higher values of log K_OW_ will be excessively hydrophobic to dissolve efficiently in water, leading to reduced bioavailability, increased soil adsorption, and a higher risk of bioaccumulation. Conversely, substances with very low values will be overly hydrophilic, making them less effective and more prone to leaching, which increases the risk of groundwater contamination. Usually, highly hydrophobic active ingredients can be transformed into salts to increase their water solubility; however, such actions result in a significant reduction of log K_OW_. Apparently, 4 orders of magnitude change in the case of MCPA can contribute to significantly hindered bioavailability. For instance, Log Kow of GB in zwitterionic form, MCPA, and MCPA as potassium salt is equal to −3.1, 3.3, and −1.3, accordingly. The calculated log K_OW_ values for functionalized alkylating agents and synthesized functionalized esterquats are presented in Table 1. The gathered data indicate that the conjugation of MCPA with GB results in compounds characterized by excellent water solubility and a wide range of log K_OW_ values, from 0.1 to 4.0, which is strongly influenced by the length of the alkyl spacer. Intriguingly, in comparison to esterquats, the functionalized alkylating agents exhibited log K_OW_ values approximately 4 orders of magnitude higher. These hydrophobic compounds demonstrate a high potential for accumulation in living organisms as they preferentially distribute into lipid-rich tissues. Therefore, the balance between hydrophobicity and hydrophilicity is a crucial factor influencing both bioavailability and environmental fate, positioning these compounds as promising candidates for further development in agrochemical applications.

Analyzing the stability of the obtained functionalized esterquats in aqueous solutions is crucial, as it provides valuable insights into whether they represent stable biological forms or a novel system that facilitates the controlled release of a biologically active moiety. Typically, esterquats exhibit stability under mildly acidic conditions; however, they undergo rapid hydrolysis, which can be significantly accelerated by an increase in the pH of the solution. In some cases, even at neutral pH, esterquats remain unstable due to micellar catalysis, which markedly enhances the rate of hydrolytic degradation.^10,24^ An advantageous feature of our concept lies in the ability of the products to acidify water during hydrolysis. This property effectively stabilizes the system after initial decomposition, significantly enhancing its long-term stability and performance. The stability of the synthesized esterquat **8** was evaluated under two different pH conditions, as shown in Figure 3.

**Figure 3:**
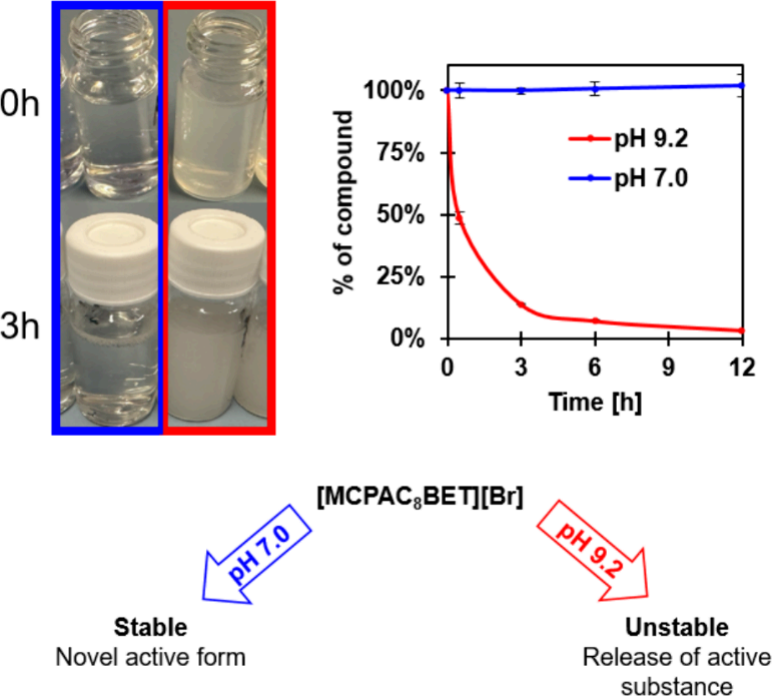
Stability of functionalized esterquat **8** in water at neutral and alkaline pH.

In neutral aqueous solution (pH 7), the tested esterquat exhibited high stability, demonstrating its resistance to hydrolytic degradation under neutral pH conditions. However, at an alkaline pH of 9.2, the compound underwent hydrolysis, yielding MCPA ester with a terminal hydroxyl group and a GB hydrobromide salt. This pH-dependent degradation suggests that under alkaline conditions ester bond cleavage occurs rapidly (85% degradation within 3 h), leading to the release of the herbicidal component in its modified form. This pH-dependent behavior underscores the potential of such systems for the controlled release of the biological moiety, which can be finely tuned through pH adjustments in an alkaline environment.

Greenhouse studies allowed us to conclude that despite their high stability in a neutral environment, the synthesized compounds retained biological activity. In tests on dicotyledonous plants, such as white mustard, they exhibited a performance comparable to the commercial product (see Table S4 and Figure S51 in the Supporting Information). This suggests that the herbicidal activity of MCPA remains intact despite its coupling with GB. We hypothesize that esterquats function as novel active substances, and that through appropriate pH adjustments, we can control the release of MCPA. The amphiphilic nature of esterquats, their interaction with plant cuticles and cellular membranes, may additionally enhance the absorption and systemic distribution of the active components, potentially maintaining or even improving the herbicidal effect.^18^

The relatively straightforward access to a new class of ionic liquids (ILs), where a biologically active anion (MCPA herbicide) is covalently linked to the GB structure via an ester bond, paves the way for the innovative modifications of well-established bioactive molecules. The observed pH-dependent hydrolysis not only highlights the potential of these synthesized compounds as novel active ingredients but also suggests their application in controlled-release formulations where degradation can be triggered by specific environmental conditions. This mechanism can lead to sustained herbicidal activity over time, thus, enhancing the efficacy and longevity of the active component. Moreover, this approach offers the flexibility to tailor critical properties such as bioavailability and release rate while mitigating common challenges like high volatility and poor water solubility of active ingredients. To the best of our knowledge, this is the first demonstration of GB being used as a molecular scaffold to incorporate a biologically active anionic agent into the cationic structure of ILs. Beyond the herbicidal applications discussed here, the presented strategy could be extended to a wide range of bioactive compounds as well as to other betaine-based derivatives. Analogous functionalized alkylating agents could potentially be used to modify antimicrobial agents (such as penicillins) or anti-inflammatory drugs (such as ibuprofen), creating novel derivatives with altered pharmacokinetic profiles or synergistic interactions between components. We are confident that this approach holds significant promise for the development of multifunctional compounds, which can offer improved efficacy against herbicide-resistant weeds or drug-resistant pathogens, thus bridging multiple scientific disciplines.

**Safety.** All experiments involving chemical synthesis were conducted in accordance with institutional safety protocols. Dibromoalkanes were handled in a well-ventilated fume hood while wearing appropriate personal protective equipment, including gloves and safety goggles, due to their potential toxicity and alkylating activity. Acetonitrile and methanol, both flammable and toxic solvents, were used with care to avoid inhalation and skin contact, and waste solvents were collected in designated containers for proper disposal. Silica gel, used for purification, was handled carefully to minimize inhalation of dust particles. No unexpected or unusually high hazards were encountered during the course of this study.

## Supplementary Material



## Data Availability

All underlying data available in the article itself and its Supporting Information.

## Notes

List of used materials, full experimental methods with detailed protocol of MCPA purification, synthesis of MCPA potassium salt, synthesis and purification of functionalized alkylating agents (with solubility in various solvents and retardation factor values) and synthesis and purification of functionalized betaine esters salts. NMR, FT-IR and MS spectra of synthesized compounds and phytotoxicity results on white mustard after foliar application (PDF)

## Abbreviations

ILs: Ionic liquids
MCPA: 4-chloro-2-methylphenoxyacetic
API: Active pharmaceutical ingredient
HILS: Herbicidal ionic liquids
GB: Glycine betaine
ACN: Acetonitrile
MeOH: Methanol
FT-IR: Fourier-transform infrared
NMR: Nuclear Magnetic Resonance
MS: Mass spectrometry
DMSO: Dimethyl sulfoxide
RPM: Revolutions per minute
DMF: *N*,*N*-dimethylformamide
K_OW_: Octanol–water partition coefficient
