# Supplementary material for: Glycine Betaine-Functionalized Ionic Liquids: Design of Versatile Bioplatform for Incorporating Biologically Active Moieties into Cations
Source: ACS Med Chem Lett. 2025 Aug 19;16(9):1756–61. doi: 10.1021/acsmedchemlett.5c00280 (PMC12434533; doi:10.1021/acsmedchemlett.5c00280)
Supplement: Supplementary file 1 [file ml5c00280_si_001.pdf]

Electronic Supplementary Information

Glycine Betaine-Functionalized Ionic Liquids: Design of Versatile Bioplatform for Incorporating Biologically Active Moieties into Cations

Witold Stachowiak,[a] Marcin Wysocki,[b] Lorenzo Guazzelli,[c] and Michał Niemczak*[a]

[a]Faculty of Chemical Technology, Poznan University of Technology, Poznan 60-965, Poland.

[b]Chair and Department of Inorganic and Analytical Chemistry, Poznan University of Medical Sciences, Poznan 60-806, Poland.

[c]Department of Pharmacy, University of Pisa, 56126 Pisa, Italy

*michal.niemczak@put.poznan.pl

Table of Contents

1. Table S1	....	S2
2. Materials	....	S4
3. Methods	....	S4–S8
4. NMR spectra	....	S9–S21
5. FT-IR spectra	....	S22–S27
6. MS spectra	....	S28–S33
7. Phytotoxicity	....	S34–S35

Table S1. Comparison of HILs with esterquat of betaine with other solutions.

Compounds	HILs with synthetic cation	HILs with betaine as cation	HILs as esterquat of choline	HILs as esterquat of betaine (this study)
General structure	Tetraalkylammonium cation + herbicidal anion 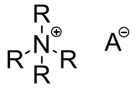	Betaine cation + herbicidal anion 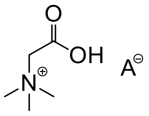	Esterquat (esterified choline) + halide or herbicidal anion 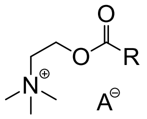	Esterquat (esterified betaine) + halide or herbicidal anion 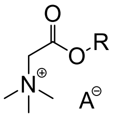
General synthesis	For commercially available QASs with tetraalkylammonium cation: ion exchange or neutralization<br><br>For commercially available amines: quaternization, then ion exchange or neutralization	Protonation of zwitterion or ion exchange in hydrochloride	R = alkyl<br>Quaternization of deanol<br><br>Functionalized compounds:<br>3 step synthesis: synthesis of acyl chlorides from herbicidal acids, esterification of deanol and quaternization	R = alkyl<br>*O*-alkylation of zwitterionic betaine<br><br>Functionalized compounds:<br>two *O*-alkylations
Weak points in synthesis	Anion exchange can sometimes be partial, difficult isolation of product from aqueous solution due to foam formation, quaternization is necessary if cation is non-available on commercial scale	Difficulty in assessment if product is present as ionic pair of binary mixture equilibrium reaction (in case of weak acid it did not lead to desired compound),	R = alkyl<br>Simple and efficient, but from fully synthetic feedstock<br><br>Functionalized compounds:<br>Multi step, complicated purification, toxic and corrosive reagents in case of functionalized compounds.	R = alkyl<br>Simple and efficient synthesis from naturally derived betaine<br><br>Functionalized compounds:<br>complicated purification
Tunability of physicochemical properties	Moderate: adjustable only by length of alkyl chains, in some cases limited to availability of QASs as substrates for ion exchange	None	R = alkyl<br>Moderate: adjustable only by length of alkyl chains<br><br>Functionalized compounds:<br>Moderate: only anion and spacer length can adjusted.	High: modifications to both headgroup, ester chain and anion
Potential ecotoxicity	Generally high or very high due to utilization of fully synthetic QASs cations known for their high toxic effect toward living organisms	Depends only from the structure of active agent	Medium or low, depending mainly on the structure of active agent and length of alkyl chains	Lack of data (potentially low)
Biodegradability	Persistent cation, low abiotic degradation	High	Enhanced biodegradability via ester bonds	Lack of data (potentially facilitated biodegradability due

				to presence of ester bonds)
Herbicidal activity	Usually comparable or better than commercial formulations, mainly due to presence of surface active cations			
Main advantages in application	Ease of synthesis, tunability and Surfactant-like behavior (eliminates use adjuvants), cheap reactants	Use of renewable cheap cation, moderate ecotoxic profile	Surfactant-like behavior (eliminates use adjuvants), some biobased origin	Green, tunable, biobased origin, surfactant-like behavior (eliminates use adjuvants), cheap reactants
Main drawbacks in application	Low biodegradability, high ecotoxicity, potential to bioaccumulation in environment	Low tunability, lower activity due to lack of amphiphilic cation	Expensive reactants, multi-step and costly synthesis with toxic reagents	Synthesis and purification require further optimization

References

1. Wilms, W.; Wozniak-Karczewska, M.; Syguda, A.; Niemczak, M.; Ławniczak, Ł.; Pernak, J.; Rogers, R. D.; Chrzanowski, Ł. Herbicidal Ionic Liquids: A Promising Future for Old Herbicides? Review on Synthesis, Toxicity, Biodegradation, and Efficacy Studies. *J. Agric. Food Chem.* **2020**, *68* (39), 10456–10488. https://doi.org/10.1021/acs.jafc.0c02894.
2. Parus, A.; Homa, J.; Radoński, D.; Framski, G.; Woźniak-Karczewska, M.; Syguda, A.; Ławniczak, Ł.; Chrzanowski, Ł. Novel Esterquat-Based Herbicidal Ionic Liquids Incorporating MCPA and MCPP for Simultaneous Stimulation of Maize Growth and Fighting Cornflower. *Ecotoxicol. Environ. Saf.* **2021**, *208*, 111595. https://doi.org/https://doi.org/10.1016/j.ecoenv.2020.111595.
3. Wysocki, M.; Stachowiak, W.; Smolibowski, M.; Olejniczak, A.; Niemczak, M.; Shamshina, J. L. Rethinking the Esterquats: Synthesis, Stability, Ecotoxicity and Applications of Esterquats Incorporating Analogs of Betaine or Choline as the Cation in Their Structure. *Int. J. Mol. Sci.* **2024**, *25* (11). https://doi.org/10.3390/ijms25115761.
4. Stachowiak, W.; Olejniczak, A.; Rzemieniecki, T.; Smolibowski, M.; Wysokowski, M.; Jesionowski, T.; Mezzetta, A.; Guazzelli, L.; Niemczak, M. Mono- and Dicationic Quaternary Ammonium Salts from Glycine Betaine: Are They Less Ecotoxic Than Currently Applied Commercial Cationic Surfactants? *ACS Sustain. Chem. Eng.* **2024**, *12* (50), 18187–18199. https://doi.org/10.1021/acssuschemeng.4c07208.
5. Stachowiak, W.; Smolibowski, M.; Kaczmarek, D. K.; Rzemieniecki, T.; Niemczak, M. Toward Revealing the Role of the Cation in the Phytotoxicity of the Betaine-Based Esterquats Comprising Dicamba Herbicide. *Sci. Total Environ.* **2022**, *845*. https://doi.org/10.1016/j.scitotenv.2022.157181.

2. Materials

Silica gel 60 and TLC plates (Supelco, Silica gel 60 with UV indicator F_{254} on aluminum) were purchased from Merck (Darmstadt, Germany), 2-methyl-4-chlorophenoxyacetic acid (95%), activated carbon (pure), betaine in zwitterionic form (98%), 1,4-dibromobutane (99%), 1,6-dibromohexane (96%), 1,8-dibromooctane (98%), 1,10-dibromodecane (97%), 1,12-dibromododecane (98%) were purchased from Sigma-Aldrich (Saint Louis, MO, USA), potassium hydroxide (87 %), acetone (99%), acetonitrile (99%), chloroform (99%), dimethyl sulfoxide (99%), N,N-dimethylformamide (99%), ethyl acetate (99%), hexane (99%), isopropanol (99%), methanol (99.8%) and toluene (99%) were obtained from Avantor (Gliwice, Poland). For chromatography, hexane was distilled prior to use to eliminate high-boiling-point impurities.

3. Methods

3.1. General

^{1}H NMR spectra were recorded on a Varian VNMR-S 400 MHz spectrometer (Palo Alto, USA) with TMS as the internal standard. ^{13}C NMR spectra were obtained with the same instrument at 100 MHz. The IR spectra were collected by using a EasyMax 102 semi-automated system (Mettler Toledo, Switzerland) connected to a ReactIR iC15 (Mettler Toledo, Switzerland) probe equipped with an MCT detector and a 9.5 mm AgX probe with a diamond tip. The data were sampled from 3000 to 650 cm^{-1} with 8 cm^{-1} resolution and processed by iCIR 4.3 software. Mass spectra were collected with Q-TOF 6546 mass spectrometer manufactured by Agilent Technologies (Santa Clara, CA, USA). Agilent Dual Jet Stream Technology Ion Source ESI operated in positive mode. The following parameters of the source were set as follows: drying gas temperature, 320°C; drying gas flow, 10 l·min^{-1}, nebulizer pressure: 45 psi, sheath gas temp: 350°C, sheath gas flow: 12 dm^{3}·min^{-1}, capillary entrance voltage 3500 V, nozzle voltage 0 V, fragmentor voltage 100 V. The water content in all obtained products was measured with a TitroLine 7500 KF trace apparatus (SI Analytics, Germany) using the Karl Fischer titration method. First, each compound was dissolved in dehydrated methanol. The water content was determined in the pure methanol as well as in the obtained methanolic solutions. Then, the water content in pure products was calculated. Log K_{ow} and the water solubility, logarithm of the octanol-water partition coefficient (Log K_{OW}), used in this study were evaluated using programs implemented in EPI (Estimation Programs Interface) Suite 4.11. EPI Suite™ is a Windows ®-based set of physical/chemical property and environmental fate estimation program developed

by US EPA. Water solubility was predicted using KOWWIN v1.68, Log K_{OW} was predicted using WSKOWWIN v1.43.

To investigate the primary hydrolysis rate, 1.5 cm^{-3} samples were collected from glass vials stored in the dark. For each pH condition, three replicates were analyzed: pH 7 (solution in demineralized water) and pH 9.2 (buffered solution in a Carbonate-Bicarbonate Buffer, prepared by dissolving 7.645 g of sodium bicarbonate and 0.954 g of anhydrous sodium carbonate in 1 dm^3 of water). Samples were taken at 3h intervals over a 12h period. The initial samples were collected after the solubilization of reagents and were thus treated as if assessed 30 minutes later. The collected samples were filtered through 0.2 µm polytetrafluoroethylene membrane syringe filters (MACHEREY-NAGEL GmbH & Co. KG, Dueren, Germany), diluted in acetonitrile and analyzed qualitatively and quantitatively in 3 replicates for esterquat 8 and its primary degradation products using ultra-high-performance liquid chromatography coupled with mass spectrometry (UHPLC-MS/MS). The primary hydrolysis analysis was conducted using an Agilent 1290 Infinity II chromatographic system coupled with a Q-TOF 6546 mass spectrometer (Agilent Technologies, Santa Clara, CA, USA). A 1 µL sample was injected into a ZORBAX RRHD Eclipse Plus C18 column (95 Å, 1.8 µm, 2.1 × 50 mm, Agilent Technologies, Santa Clara), maintained at 35°C. The mobile phase consisted of 10mM ammonium formate in water, pH=3 (solution A) and acetonitrile (solution B). A gradient elution was applied, increasing from 5% to 95% of B over 5 minutes. The Agilent Dual Jet Stream Technology ion source operated in positive electrospray ionization (ESI) mode. The following source parameters were set: drying gas temperature, 320°C; drying gas flow rate, 10 dm^3/min; nebulizer pressure, 45 psi; sheath gas temperature, 350°C; sheath gas flow rate, 12 dm^3/min; capillary entrance voltage, 4000 V; nozzle voltage, 0 V; and fragmentor voltage, 100 V.

3.2. Synthesis methodology

3.2.1. Purification of MCPA

First, 2-methyl-4-chlorophenoxyacetic acid (MCPA, m.p. 114.0-118.0 °C) was purified prior to use by double recrystallization from toluene combined with adsorption of impurities on activated carbon. MCPA (200 g) was placed in 1000 cm^3 flask equipped with magnetic stirrer with 525 cm^3 of toluene and 10 g of activated carbon. The mixture was then heated to 110 °C and stirred for 30 minutes. Subsequently, the mixture was filtered at 70 °C and the remaining filtrate was cooled in room temperature and then in the refrigerator until 4 °C temperature was reached. The crystallized product was subjected to procedure once again, and was then dried

under reduced pressure (60 °C, 40 mbar, 4 h) to obtain 126 g of white crystals (m.p. 117.9-118.5 °C, for NMR see fig. S2-S3, yield equal to 63%).

3.2.2. Synthesis of potassium salt of MCPA

The purified MCPA (50 g) was placed in 250 cm^3 flask with 100 cm^3 of isopropanol and 5 cm^3 of methanol and mixed at 40 °C until dissolved. The mixture was then poured into another 500 cm^3 flask containing 13.98 g of potassium hydroxide dissolved in 120 cm^3 of isopropanol and 6 cm^3 of methanol at 40 °C. The leftover solution was rinsed with 10 cm^3 of isopropanol and poured into the main solution. The mixture was then mixed for 10 minutes and the precipitate was filtered under reduced pressure and subsequently dried in 65 °C for 4 hours yielding 59 g (99%) of crude product.

3.2.3. Synthesis and purification of functionalized alkylating agents

In 100 cm^3 flask equipped with magnetic stirrer placed 7.16 g (30 mmol) of potassium-MCPA, 30 cm^3 of methanol and acetonitrile (1:1 v/v) and three-fold stoichiometric excess amount of respective 1,ω-dibromoalkanes, and mixed for 24-48h at 60 °C under reflux. Subsequently, the reaction mixture was cooled at ambient temperature, concentrated in rotary evaporator, and the inorganic precipitate was filtered out and rinsed with acetonitrile (2 x 10 cm^3). The filtrate was then concentrated on rotary evaporator. Concentrate was subjected to vacuum distillation to remove excess of 1,ω-dibromoalkanes as well as volatile impurities (heating mantle heating to 220°C and 4 mbar vacuum were sufficient for **1**, **2** and **3**. For **4** and **5**, 0.3 mbar vacuum was used).

The obtained crude products **1-2** were purified by vacuum distillation (0.3 mbar, colourless fraction of product were collected to 80°C, later orange liquids starts to condense), while purification of **3-5** was performed using flash chromatography (ethyl acetate:hexane 1:9 v/v on 60 mesh silica gel, 0.4 bar overpressure). Products were identified with TLC plates (Silica gel 60 with UV indicator F_{254} on aluminum) eluted by 1:9 ethyl acetate:hexane. Retardation factor of products 1-5 were summarized in table S2. It should be noted, that any residual amount of 1,ω-dibromoalkanes should be wash out as they are characterized by Rf>0.95 in any used system, but most of them must be distilled before chromatography, due to the fact that they greatly increase eluting power of hexane.

Table S2. Retardation factor of **1-5** on silica gel 60.

		Retardation factor		
	**Eluent**	**Hexane**	**1:19 v:v ETAC[1]:Hexane**	**1:9 v:v ETAC:Hexane**
**No**	**Compound**			
**1**	MCPAC_4Br	0	0.15	0.28
**2**	MCPAC_6Br	0	0.19	0.33
**3**	MCPAC_8Br	0	0.25	0.39
**4**	MCPAC_{10}Br	0	0.27	0.46
**5**	MCPAC_{12}Br	0	0.33	0.54

[1] ETAC: ethyl acetate

During optimization different solvents ant their mixtures in various ratios were investigated. The tested systems include methanol, dimethylformamide, dimethylsulfoxide and mixtures of methanol with acetonitrile in three different ratios: 3:1, 1:1 and 1:3, respectively. Starting with methanol, the process resulted in low yields, despite good solubility of the substrates. Additionally, moderate solubility of by-forming potassium bromide provided additional purification challenges. Carrying the process in dimethylsulfoxide appeared to favor alkylation of the solvent instead of the substrate. Considering the structure of dimethylsulfoxide, the most possible outcome is associated with alkylation of the central sulfur atom (J. Forrester, R. V. H. Jones, P. N. Preston and E. S. C. Simpson, *J. Chem. Soc. Perkin Trans. 1*, 1995, 2289–2291.), although the thorough analysis of the occurring processes was given up since the reaction mixture appeared to be highly irritating. On the other hand, equal volume mixture of methanol with acetonitrile revealed combined benefits of both solvents: good solubility of reagents (crucial due to critically low solubility of MCPA potassium salt in acetonitrile), enhancement of the reaction efficiency due to presence of polar aprotic solvent, and easier purification due to precipitation of inorganic impurities. Attempts to improve the reaction conditions were based on changing the solvents' ratio. As such, changes in this ratio resulted in enhancement of side-occurring transesterification of the obtained alkylating agents (Fig. S1), as well as decrease in the reaction rate, thus significantly lowering the yield of the desired products. Dimethylformamide can be used instead of mixture of acetonitrile:methanol with similar effect, but high boiling point of dimethylformamide make purification more complicated.

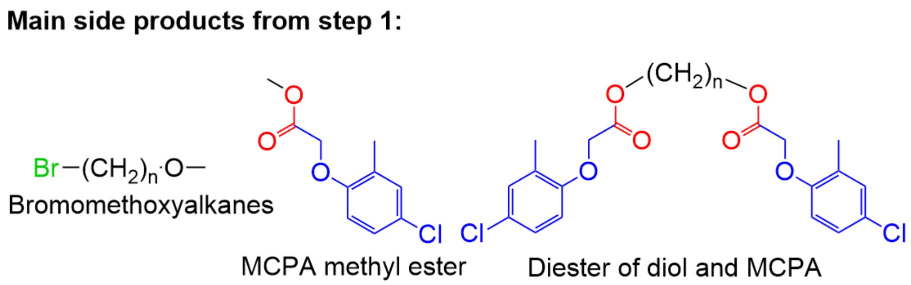

Fig. S1. Main side products formed in the step 1 of the process.

The crystallization potential of synthesized compounds **1**–**5** was also investigated. However, their solubility in various solvents (Table S3), assessed following Vogel's methodology (*A. I. Vogel, Textbook of Practical Organic Chemistry*, 5th ed., Longman, 1989), proved unfavorable for developing such a method. Therefore, only distillation and chromatography were used to purificate compounds **1-5**.

Table S3. Solubility of the alkylating agents (**1-5**) in various solvents.

**No**	**Water**	**Methanol**	**DMSO**	**Acetonitrile**	**Acetone**	**Chloroform**	**Isopropanol**	**Ethyl acetate**	**Toluene**	**Hexane**
	9.0[a]	6.6	6.5	6.2	5.4	4.4	4.3	4.3	2.3	0.0
**1**	–	+	+	+	+	+	+	+	+	+
**2**	–	+	+	+	+	+	+	+	+	+
**3**	–	+	+	+	+	+	+	+	+	+
**4**	–	+	+	+	+	+	+	+	+	+
**5**	–	+	+	+	+	+	+	+	+	+

[a] Snyder polarity index; "+" ready solubility (>0.1 g in 1 cm^3 of solvent at 25°C); "-" low solubility (<0.1 g in 3 cm^3 of solvent at 50°C)

3.2.4. Synthesis and purification of functionalized betaine esters salts

In 50 cm^3 flask 6.6 mmol (10% molar excess) of respective ω-bromoalkyl 4-methyl-2-chlorophenoxyacetate 6 mmol amount of betaine and 6 cm^3 of acetonitrile were added. The reagents were then mixed at 82 °C for 36 hours under reflux. It is crucial to ensure thorough mixing, as betaine dissolves during the reaction, and inadequate mixing may extend the reaction time. The solvent was then evaporated, subsequently the crude products were rinsed with ethyl acetate and dried at 50 °C for 4 hours under reduced pressure (0.3 mbar). Excess of ω-bromoalkyl 4-methyl-2-chlorophenoxyacetate can be later retrieved from ethyl acetate phase.

4. NMR spectra

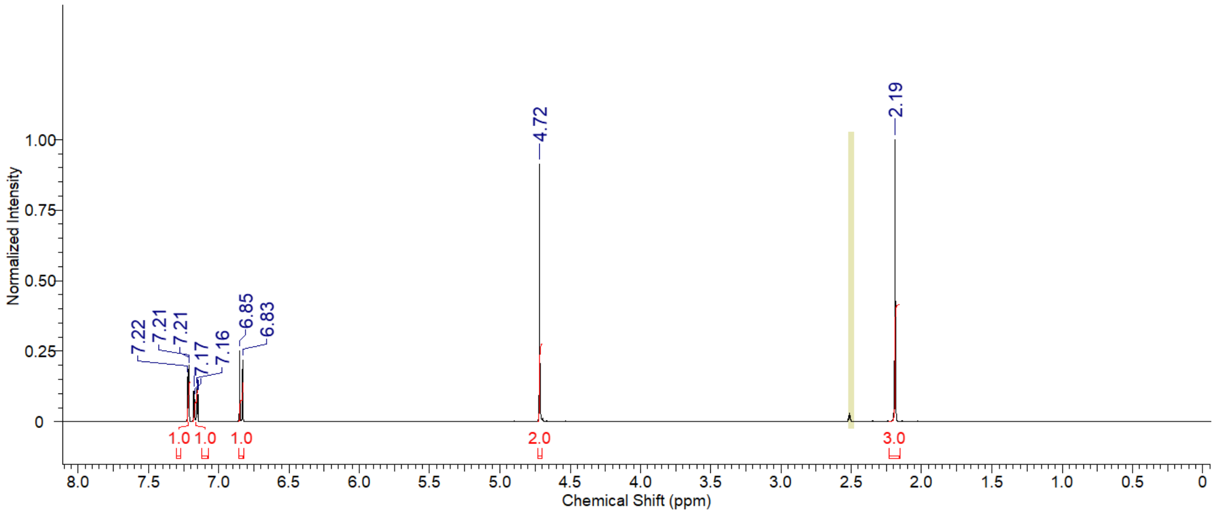

^{1}H NMR (DMSO-d_6) δ [ppm] = 2.19 (s, 3H), 4.72 (s, 2H), 6.84 (d, J=8.8 Hz, 1H), 7.16 (dd, J=8.7, 2.7 Hz, 1H), 7.22 (d, J=2.7 Hz, 1H).

Fig. S2. ^{1}H NMR of 2-methyl-4-chlorophenoxyacetic acid (MCPA acid).

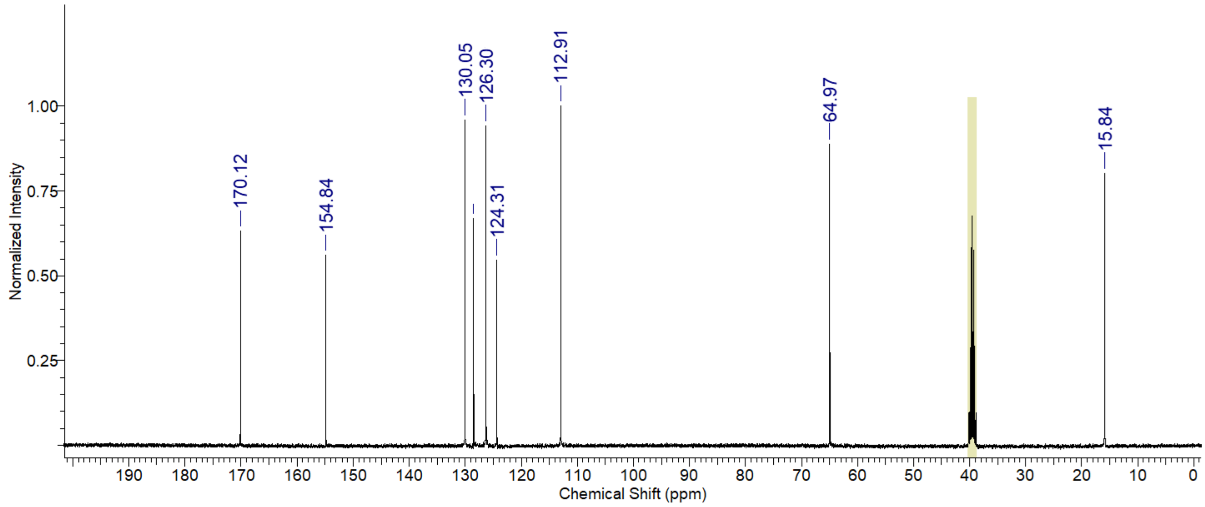

^{13}C NMR (DMSO-d_6) δ [ppm] = 15.8, 65.0, 112.9, 124.3, 126.3, 128.5, 130.1, 154.8, 170.1.

Fig. S3. ^{13}C NMR of 2-methyl-4-chlorophenoxyacetic acid (MCPA).

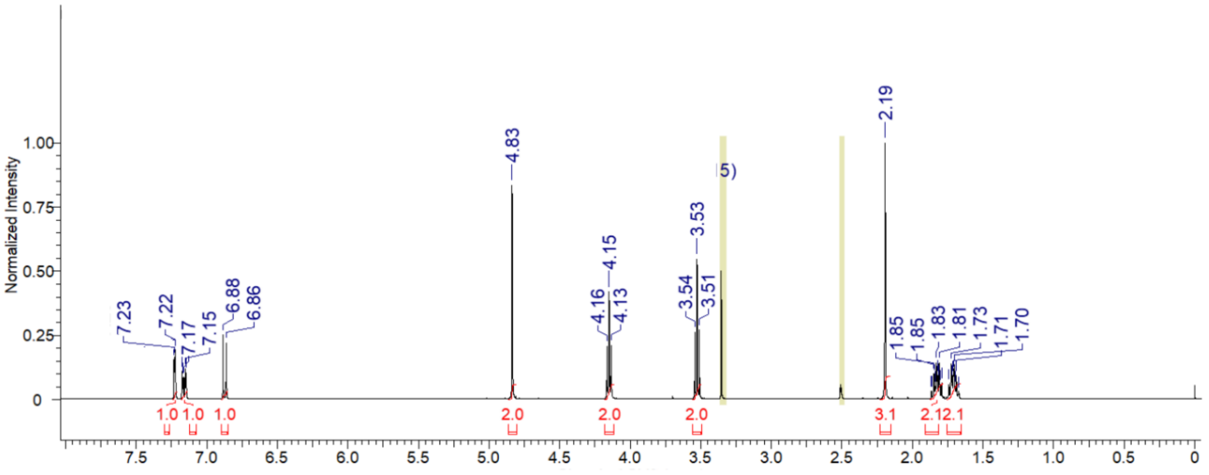

^{1}H NMR (DMSO-d_6) δ [ppm] = 1.67-1.75 (m, 2H), 1.78-1.87 (m, 2H), 2.19 (s, 3H), 3.53 (t, J=6.6 Hz, 2H), 4.15 (t, 6,4 Hz, 2H), 4.83 (s, 2H), 6.87 (d, J=8.7 Hz, 1H), 7.16 (dd, J=8.7, 2.6 Hz, 1H), 7.22 (d, J=2.7Hz, 1H).

Fig. S4. ^{1}H NMR of ω-bromobutyl 4-methyl-2-chlorophenoxyacetate (**1** - MCPAC$_4$Br).

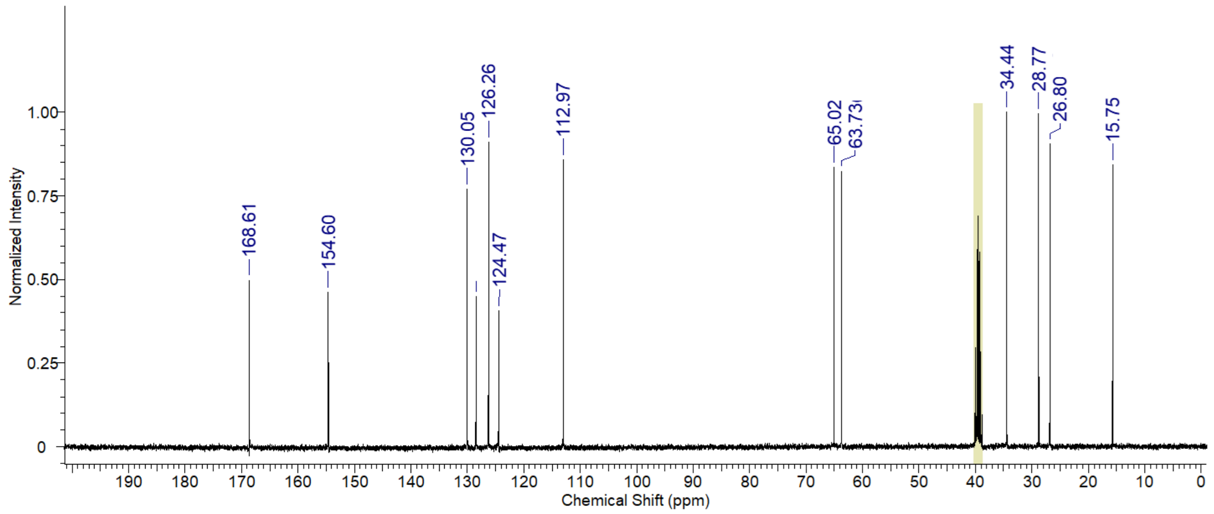

^{13}C NMR (DMSO-d_6) δ [ppm] = 15.8, 26.8, 28.8, 34.4, 63.7, 65.0, 113.0, 124.5, 126.3, 128.5, 130.0, 154.6, 168.6.

Fig. S5. ^{13}C NMR of ω-bromobutyl 4-methyl-2-chlorophenoxyacetate (**1** - MCPAC$_4$Br).

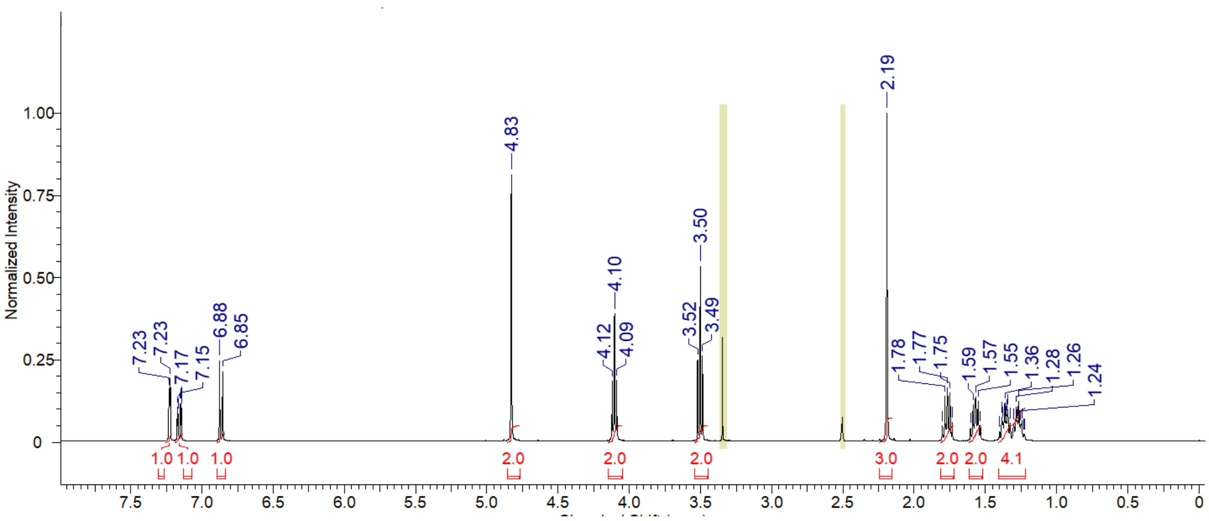

^{1}H NMR (DMSO-d_6) δ [ppm] = 1.20-1.40 (m, 4H), 1.57 (q, 2H), 1.77 (q, 2H), 2.19 (s, 3H), 3.50 (t, J=6.7 Hz, 2H), 4.10 (t, J=6.4 Hz, 2H), 4.83 (s, 2H), 6.87 (d, J=8.8 Hz, 1H), 7.16 (dd, J=8.8, 2.7 Hz, 1H), 7.23 (d, J=2.7 Hz, 1H).

Fig. S6. ^{1}H NMR of ω-bromohexyl 4-methyl-2-chlorophenoxyacetate (**2** - MCPAC$_6$Br).

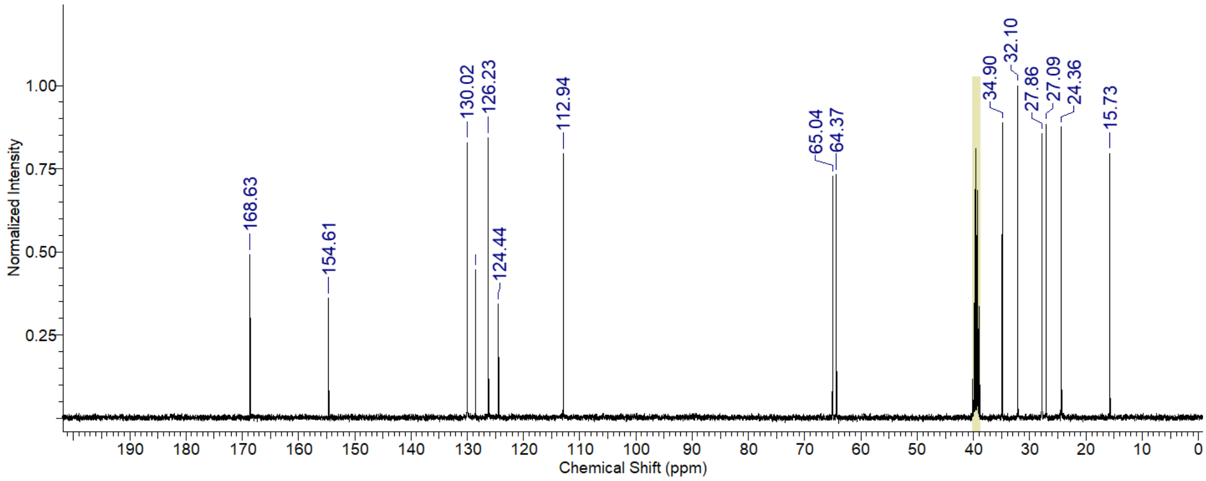

^{13}C NMR (DMSO-d_6) δ [ppm] = 15.7, 24.4, 27.1, 27.9, 32.1, 34.9, 64.4, 65.0, 112.9, 124.4, 126.2, 128.5, 130.0, 154.6, 168.6.

Fig. S7. ^{13}C NMR of ω-bromohexyl 4-methyl-2-chlorophenoxyacetate (**2** - MCPAC$_6$Br).

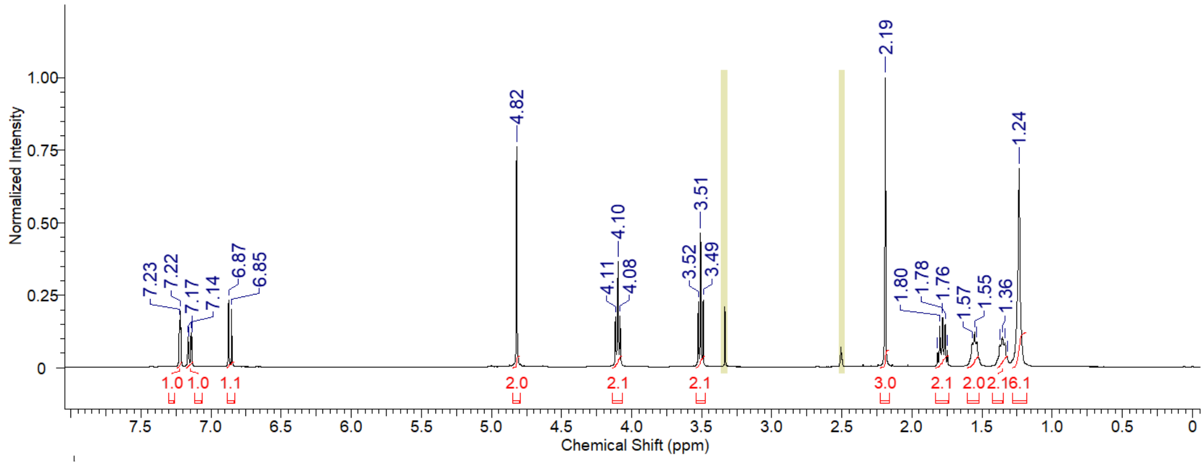

^{1}H NMR (DMSO-d_6) δ [ppm] = 1.19-1.30 (m, 6H), 1.32-1.41 (m, 2H), 1.51-1.59 (m, 2H), 1.78 (q, J=7.1 Hz, 2H), 2.19 (s, 3H), 3.51 (t, J=6.7 Hz, 2H), 4.10 (t, J=6.5 Hz, 2H), 4.82 (s, 2H), 6.86 (d, J=8.8 Hz, 1H), 7.15 (d, J=8.7 Hz, 1H), 7.22 (d, J=2.7 Hz, 1H).

Fig. S8. ^{1}H NMR of ω-bromooctyl 4-methyl-2-chlorophenoxyacetate (**3** - MCPAC$_8$Br).

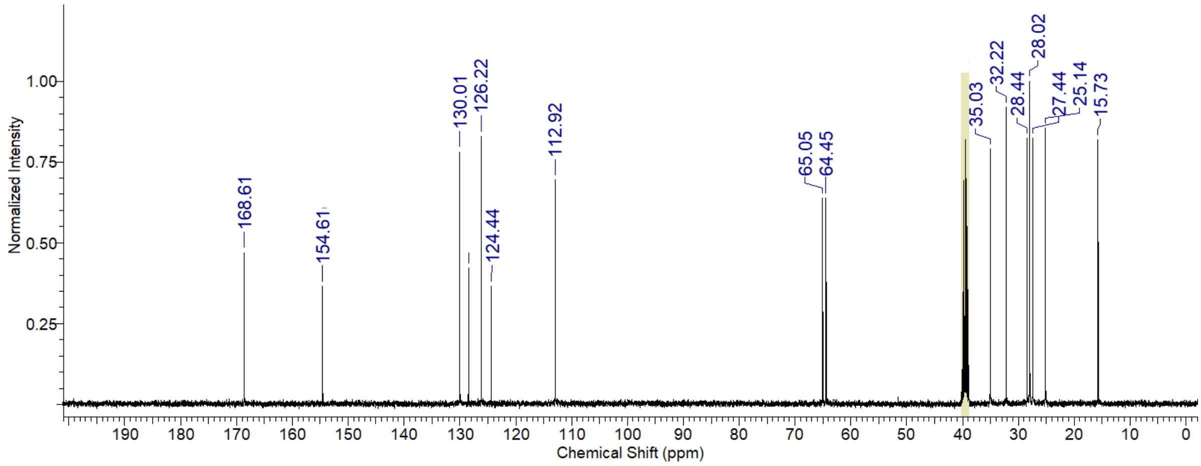

^{13}C NMR (DMSO-d_6) δ [ppm] = 15.7, 25.1, 27.4, 28.0, 28.4, 32.2, 35.0, 64.5, 65.0, 112.9, 124.4, 126.2, 128.5, 130.0, 154.6, 168.6.

Fig. S9. ^{13}C NMR of ω-bromooctyl 4-methyl-2-chlorophenoxyacetate (**3** - MCPAC$_8$Br).

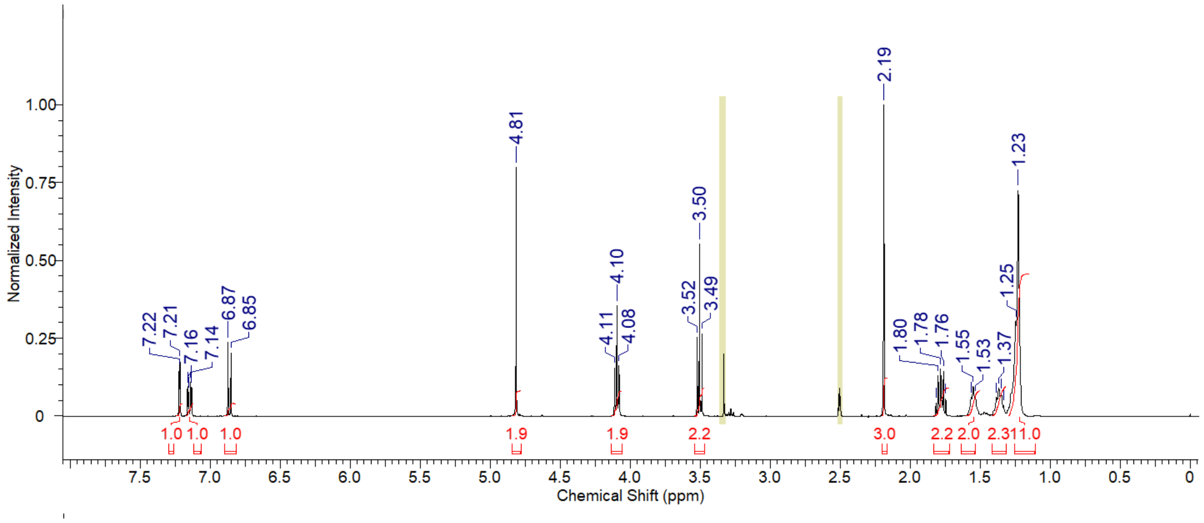

^{1}H NMR (DMSO-d_6) δ [ppm] = 1.17-1.31 (m, 10H), 1.32-1.39 (m, 2H), 1.50-1.60 (m, 2H), 1.78 (q, J=7.1 Hz, 2H), 2.19 (s, 3H), 3.50 (t, J=6.7 Hz, 2H), 4.10 (t, J=6.5 Hz, 2H), 4.81 (s, 2H), 6.86 (d, J=8.8 Hz, 1H), 7.15 (dd, J=8.6, 2.6 Hz, 1H), 7.22 (d, J=2.7 Hz, 1H).

Fig. S10. ^{1}H NMR of ω-bromodecyl 4-methyl-2-chlorophenoxyacetate (**4** - MCPAC$_{10}$Br).

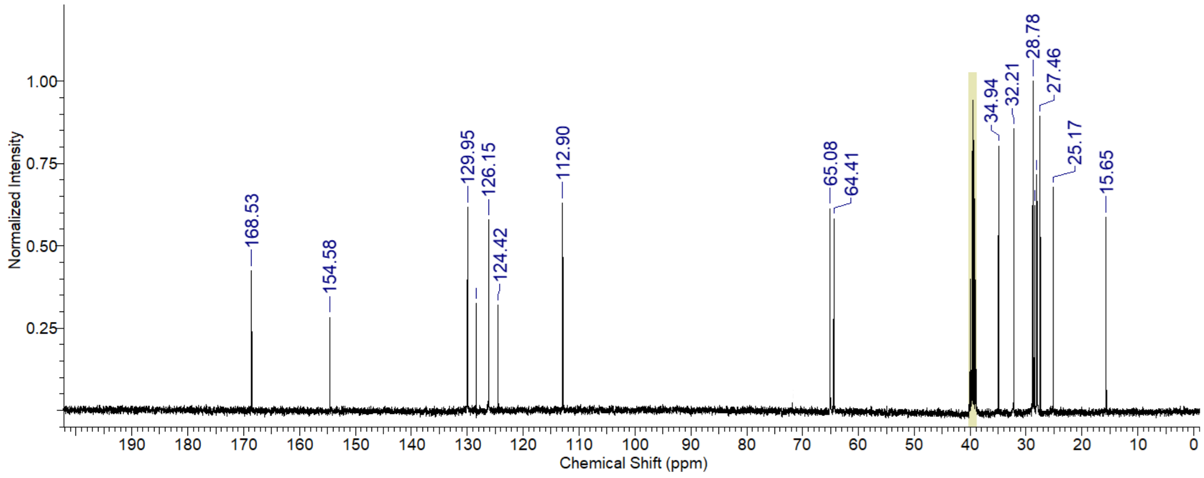

^{13}C NMR (DMSO-d_6) δ [ppm] = 15.7, 25.2, 27.5, 28.0, 28.8, 32.2, 35.0, 64.4, 65.1, 112.9, 124.4, 126.2, 128.5, 130.0, 154.6, 168.5.

Fig. S11. ^{13}C NMR of ω-bromodecyl 4-methyl-2-chlorophenoxyacetate (**4** - MCPAC$_{10}$Br).

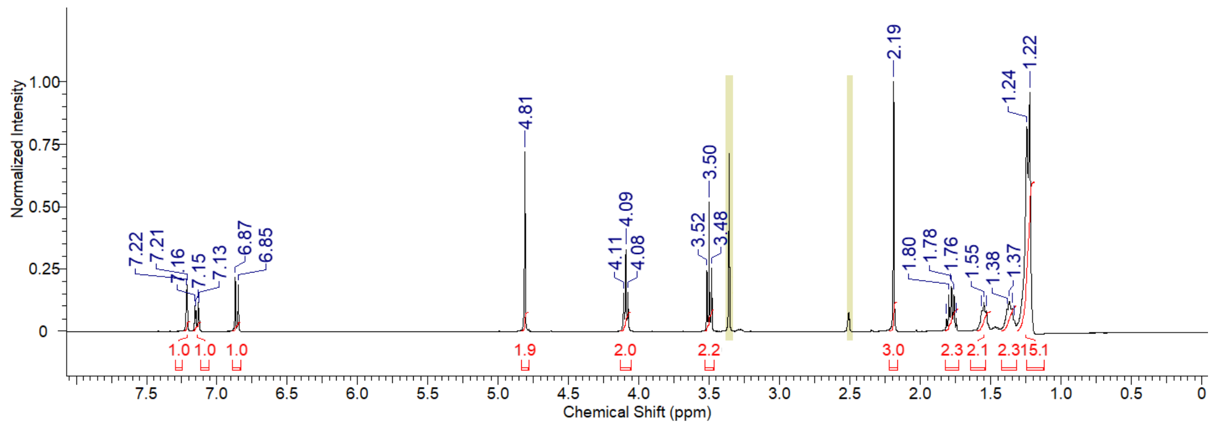

^{1}H NMR (DMSO-d_6) δ [ppm] = 1.17-1.31 (m, 14H), 1.32-1.41 (m, 2H), 1.50-1.60 (m, 2H), 1.78 (q, J=7.1 Hz, 2H), 2.19 (s, 3H), 3.50 (t, J=6.7 Hz, 2H), 4.09 (t, J=6.5 Hz, 2H), 4.81 (s, 2H), 6.86 (d, J=8.7 Hz, 1H), 7.14 (dd, J=8.7, 2.4 Hz, 1H), 7.21 (d, J=2.7 Hz, 1H).

Fig. S12. ^{1}H NMR of ω-bromododecyl 4-methyl-2-chlorophenoxyacetate (**5** - MCPAC$_{12}$Br).

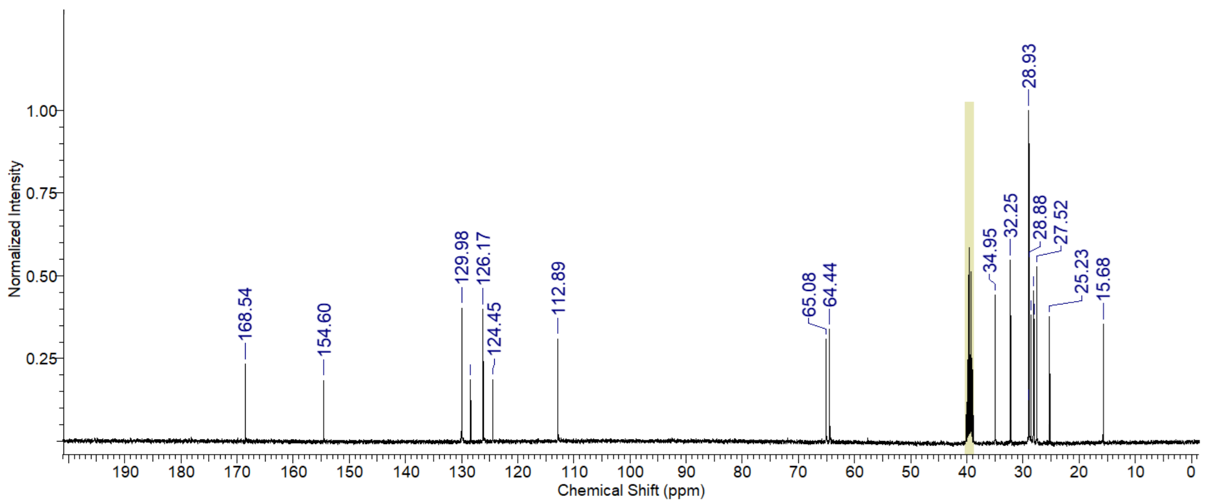

^{13}C NMR (DMSO-d_6) δ [ppm] = 15.7, 25.2, 27.5, 28.0, 28.9, 32.3, 35.0, 64.4, 65.1, 112.9, 124.5, 126.2, 128.5, 130.0, 154.6, 168.5.

Fig. S13. ^{13}C NMR of ω-bromododecyl 4-methyl-2-chlorophenoxyacetate (**5** - MCPAC$_{12}$Br).

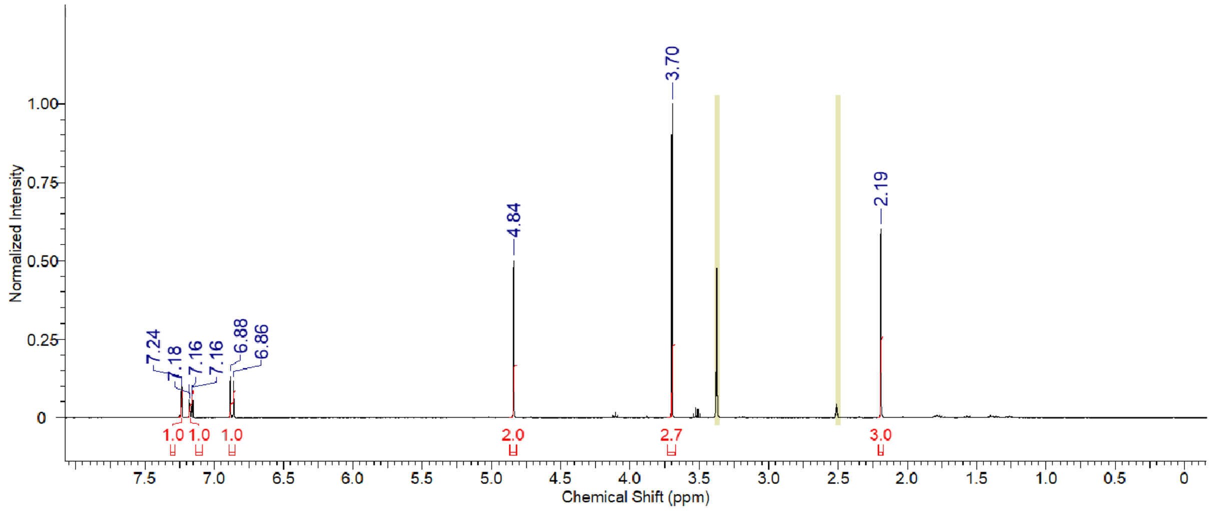

^{1}H NMR (DMSO-d_6) δ [ppm] = 2.19 (s, 3H), 3.70 (s, 3H), 4.84 (s, 2H), 6.87 (d, J=8.7 Hz, 1H), 7.17 (dd, J=8.7, 2.4 Hz, 1H), 7.24 (d, J=2.7 Hz, 1H).

Fig. S14. ^{1}H NMR of methyl 4-methyl-2-chlorophenoxyacetate (MCPACH3).

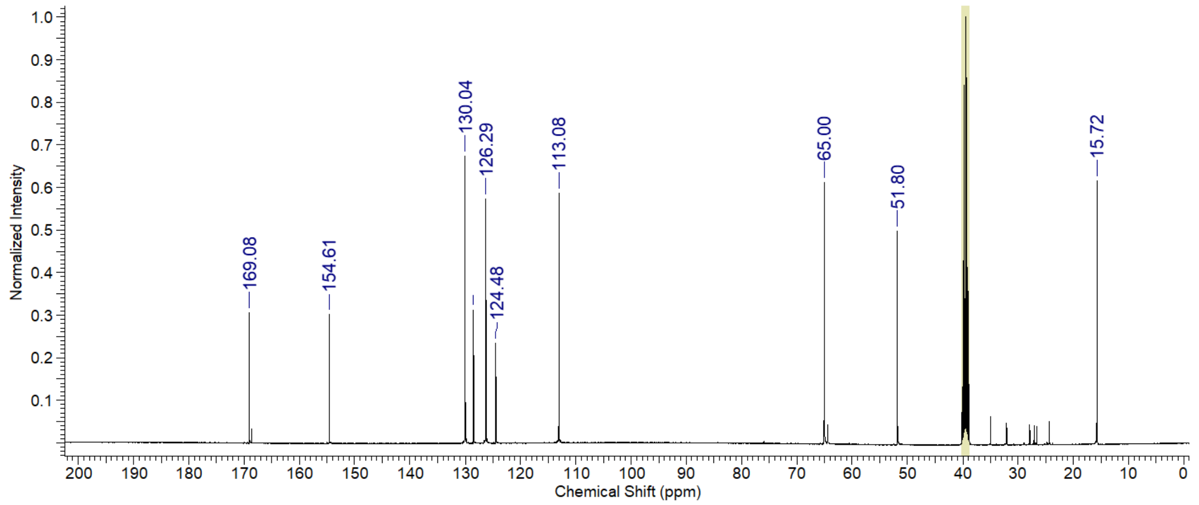

^{13}C NMR (DMSO-d_6) δ [ppm] = 15.7, 51.8, 65.0, 113.1, 124.5, 126.3, 128.5, 130.0, 154.6, 169.1.

Fig. S15. ^{13}C NMR of methyl 4-methyl-2-chlorophenoxyacetate (MCPACH3).

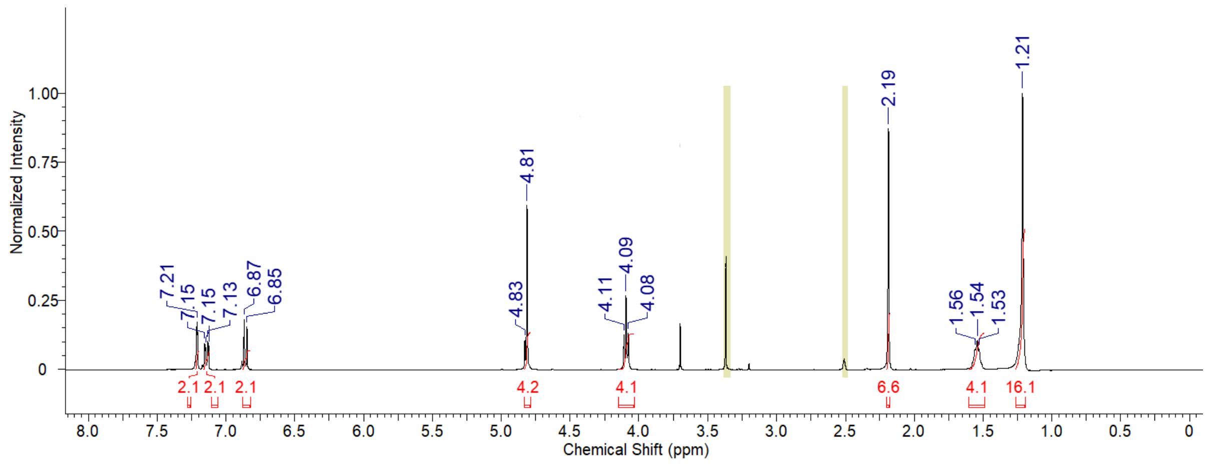

^{1}H NMR (DMSO-d_6) δ [ppm] = 1.21-1.31 (m, 16H), 1.50-1.60 (m, 4H), 2.19 (s, 6H), 4.09 (t, J=6.4 Hz, 4H), 4.81 (s, 4H), 6.86 (d, J=8.8 Hz, 2H), 7.14 (dd, J=8.7, 2.6 Hz, 2H), 7.21 (d, J=2.6 Hz, 2H).

Fig. S16. ^{1}H NMR of dodecyl di(4-methyl-2-chlorophenoxy)acetate (MCPAC_{12}MCPA).

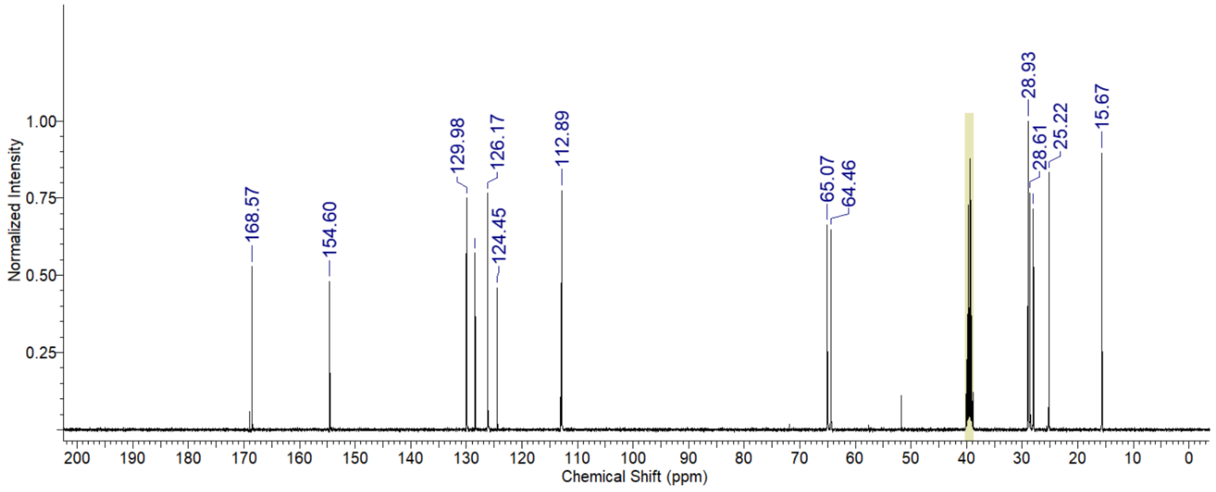

^{13}C NMR (DMSO-d_6) δ [ppm] = 15.7, 25.2, 28.0, 28.6, 28.9, 64.5, 65.1, 112.9, 124.5, 126.2, 128.4, 130.0, 154.6, 168.6.

Fig. S17. ^{13}C NMR of dodecyl di(4-methyl-2-chlorophenoxy)acetate (MCPAC_{12}MCPA).

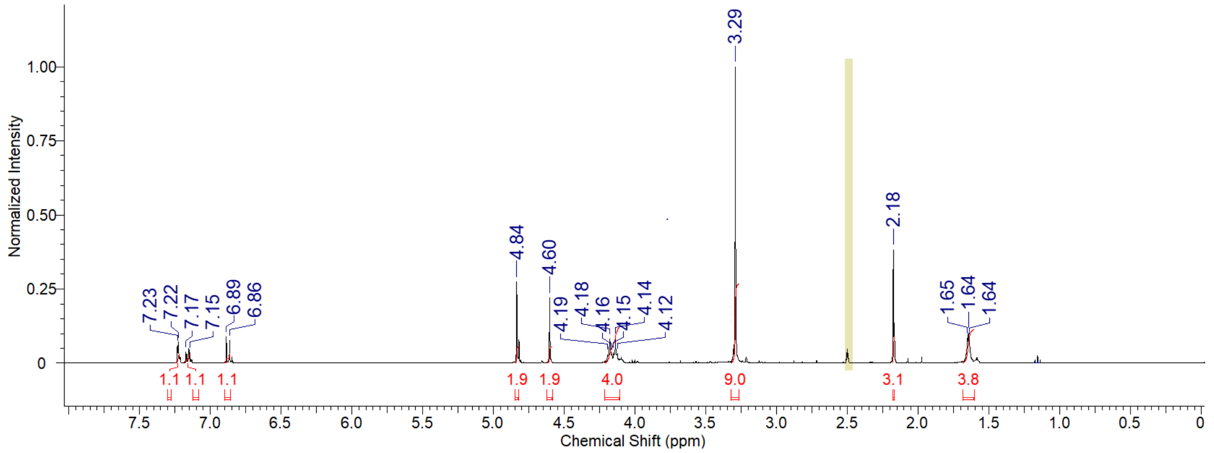

^{1}H NMR (DMSO-d_6) δ [ppm] = 1.63-1.72 (m, 4H), 2.18 (s, 3H), 3.29 (s, 9H), 4.11-4.23 (m, 4H), 4.60 (s, 2H), 4.84 (s, 2H), 6.87 (d, *J*=8.8 Hz, 1H), 7.16 (dd, *J*=8.7, 2.7 Hz, 1H), 7.23 (d, *J*=2.7 Hz, 1H).

Fig. S18. ^{1}H NMR of esterquat **6** ([MCPAC$_4$BET][Br]).

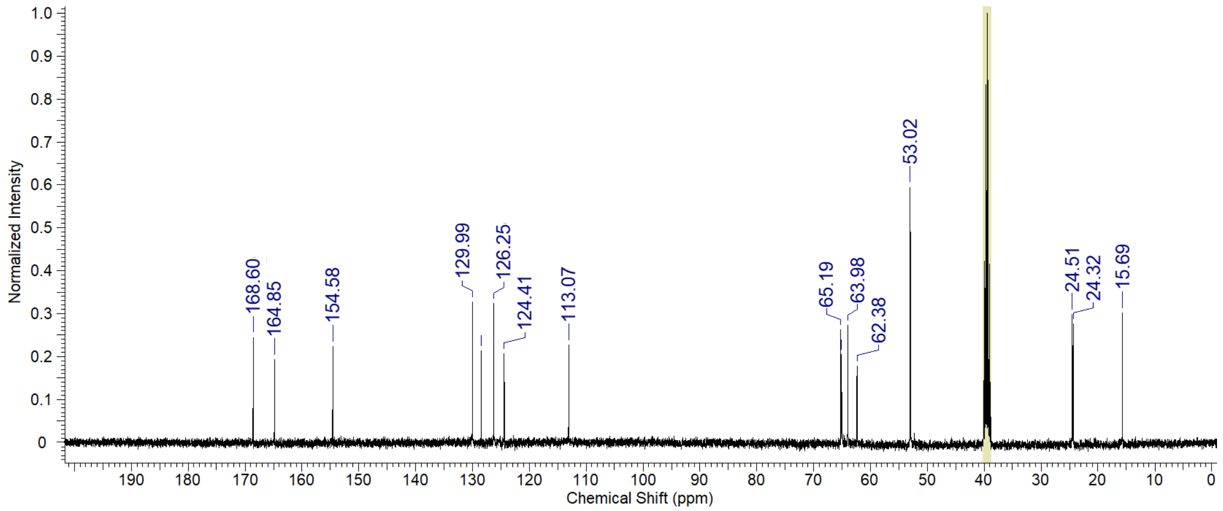

^{13}C NMR (DMSO-d_6) δ [ppm] = 15.7, 24.3, 24.5, 53.0, 62.4, 64.0, 65.2, 113.1, 124.4, 126.3, 128.5, 130.0, 154.6, 164.9, 168.6.

Fig. S19. ^{13}C NMR of esterquat **6** ([MCPAC$_4$BET][Br]).

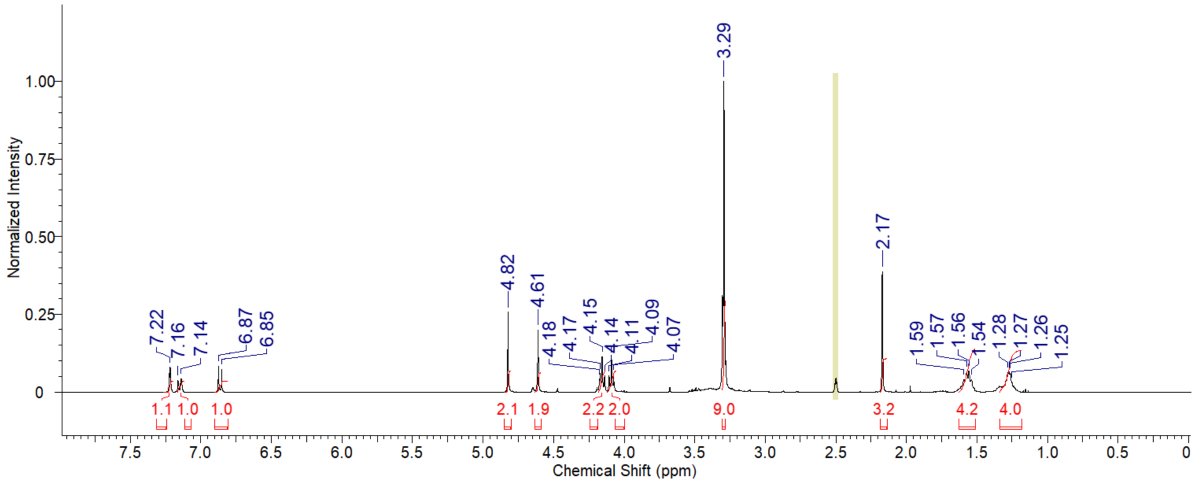

^{1}H NMR (DMSO-d_6) δ [ppm] = 1.20-1.38 (m, 4H), 1.50-1.65 (m, 4H), 2.17 (s, 3H), 3.29 (s, 9H), 4.09 (t, *J*=6.5 Hz, 2H), 4.16 (t, *J*=6.5 Hz, 2H), 4.61 (s, 2H), 4.82 (s, 2H), 6.86 (d, *J*=8.8 Hz, 1H), 7.15 (dd, *J*=8.7, 2.7 Hz, 1H), 7.22 (d, *J*=2.4 Hz, 1H).

Fig. S20. ^{1}H NMR of esterquat **7** ([MCPAC$_6$BET][Br]).

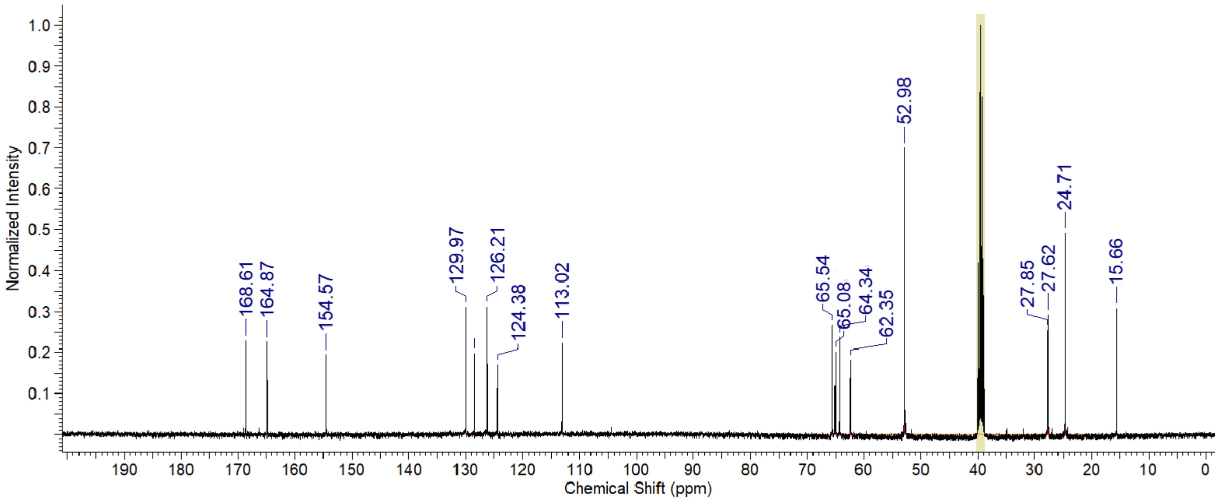

^{13}C NMR (DMSO-d_6) δ [ppm] = 15.7, 24.7, 27.6, 27.9, 53.0, 62.4, 64.3, 65.1, 65.5, 113.0, 124.4, 126.2, 128.5, 130.0, 154.6, 164.9, 168.6.

Fig. S21. ^{13}C NMR of esterquat **7** ([MCPAC$_6$BET][Br]).

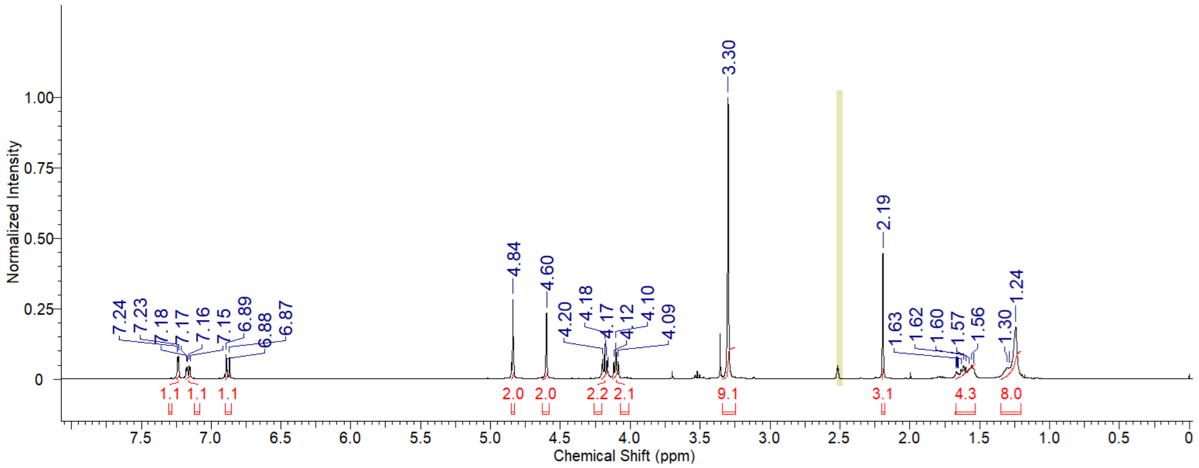

^{1}H NMR (DMSO-d_6) δ [ppm] = 1.20-1.34 (m, 8H), 1.53-1.67 (m, 4H), 2.19 (s, 3H), 3.30 (s, 9H), 4.10 (t, *J*=6.5 Hz, 2H), 4.18 (t, *J*=6.5 Hz, 2H), 4.60 (s, 2H), 4.84 (s, 2H), 6.88 (d, *J*=8.8 Hz, 1H), 7.16 (dd, *J*=8.8, 2.7 Hz, 1H), 7.24 (d, *J*=2.8 Hz, 1H).

Fig. S22. ^{1}H NMR of esterquat **8** ([MCPAC$_8$BET][Br]).

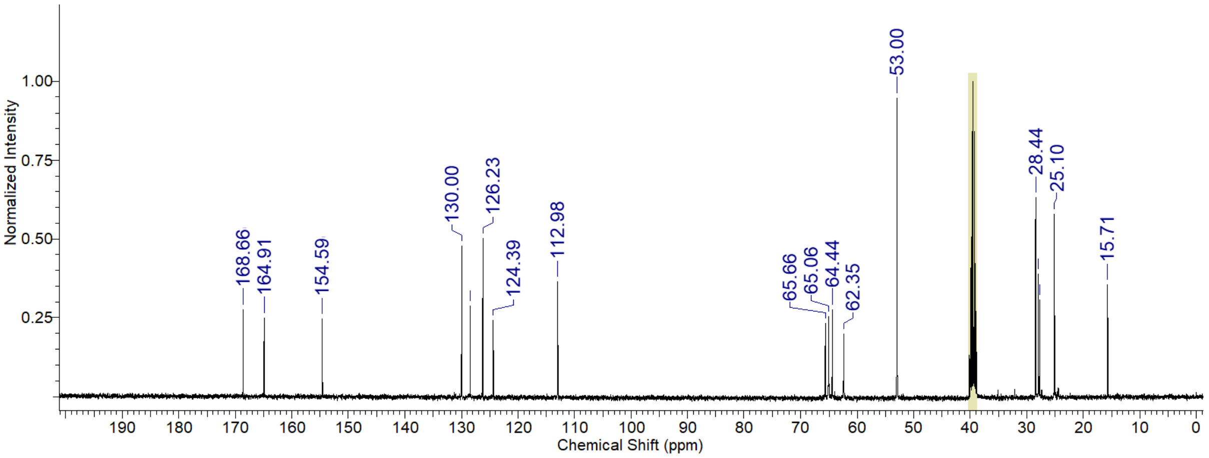

^{13}C NMR (DMSO-d_6) δ [ppm] = 15.7, 25.1, 28.0, 28.4, 53.0, 62.4, 64.4, 65.1, 65.7, 113.0, 124.4, 126.2, 128.4, 130.0, 154.6, 164.9, 168.7.

Fig. S23. ^{13}C NMR esterquat **8** ([MCPAC$_8$BET][Br]).

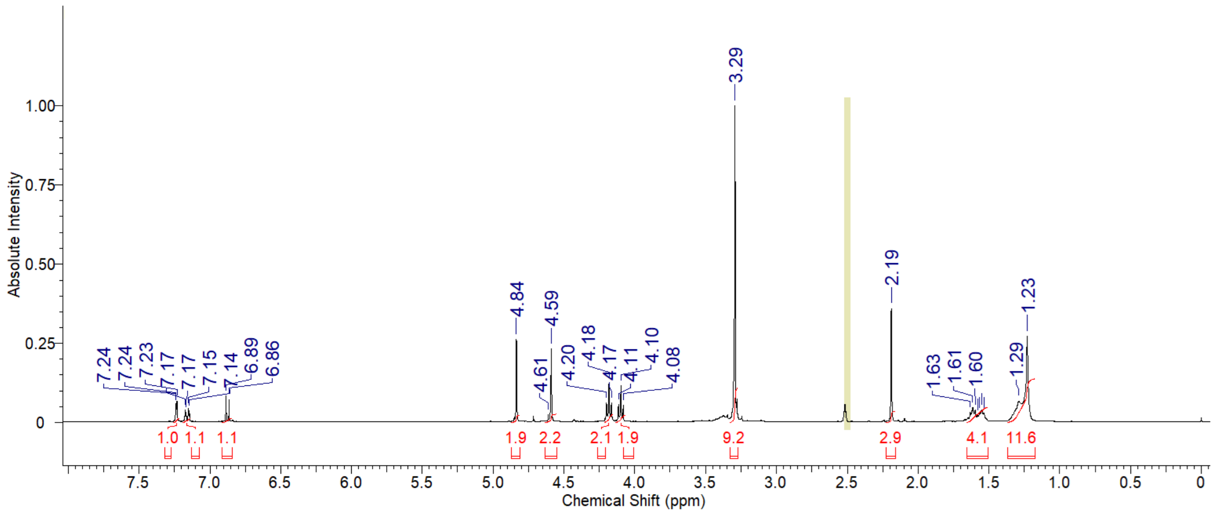

^{1}H NMR (DMSO-d_6) δ [ppm] = 1.17-1.37 (m, 12H), 1.51-1.66 (m, 4H), 2.19 (s, 3H), 3.29 (s, 9H), 4.10 (t, *J*=6.5 Hz, 2H), 4.18 (t, *J*=6.6 Hz, 2H), 4.59 (s, 2H), 4.84 (s, 2H), 6.88 (d, *J*=8.8 Hz, 1H), 7.16 (dd, *J*=8.8, 2.6 Hz, 1H), 7.24 (d, *J*=2.6 Hz, 1H).

Fig. S24. ^{1}H NMR of esterquat **9** ([MCPAC$_{10}$BET][Br]).

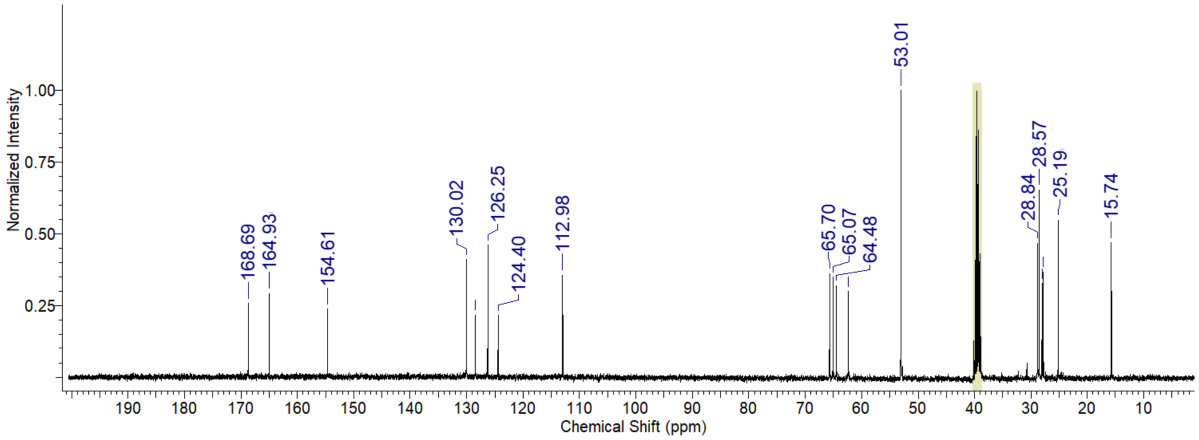

^{13}C NMR (DMSO-d_6) δ [ppm] = 15.7, 25.2, 27.8, 28.0, 28.5, 28.8, 53.0, 62.3, 64.4, 65.1, 65.7, 113.0, 124.4, 126.3, 128.4, 130.0, 154.6, 164.9, 168.7.

Fig. S25. ^{13}C NMR of esterquat **9** ([MCPAC$_{10}$BET][Br]).

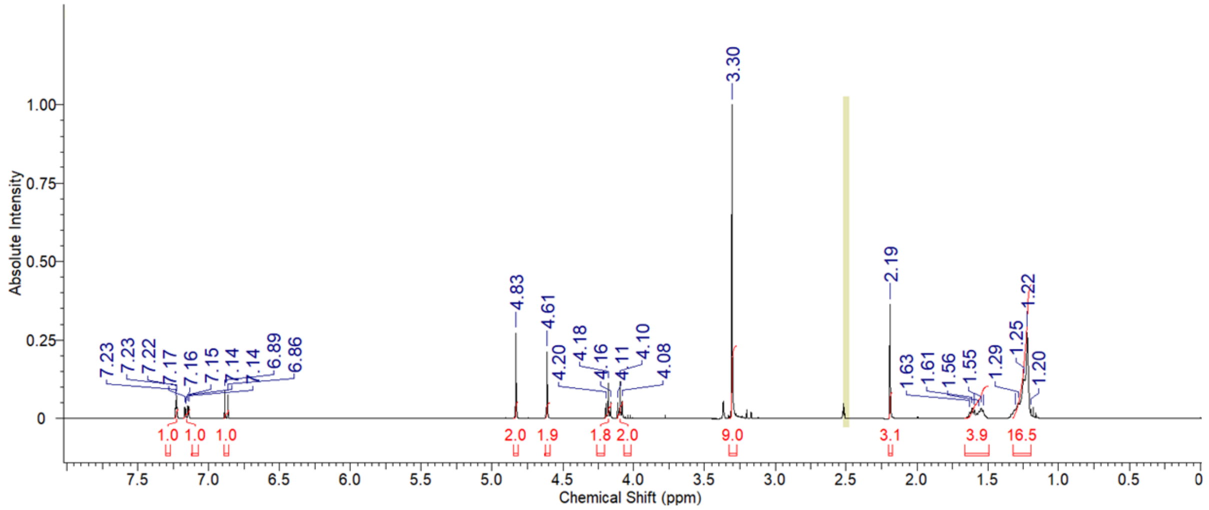

^{1}H NMR (DMSO-d_6) δ [ppm] = 1.20-1.35 (m, 16H), 1.50-1.66 (m, 4H), 2.19 (s, 3H), 3.30 (s, 9H), 4.10 (t, *J*=6.5 Hz, 2H), 4.18 (t, *J*=6.6 Hz, 2H), 4.61 (s, 2H), 4.83 (s, 2H), 6.88 (d, *J*=8.8 Hz, 1H), 7.15 (dd, *J*=8.8, 2.7 Hz, 1H), 7.23 (d, *J*=2.7 Hz, 1H).

Fig. S26. ^{1}H NMR of esterquat **10** ([MCPAC_{12}BET][Br]).

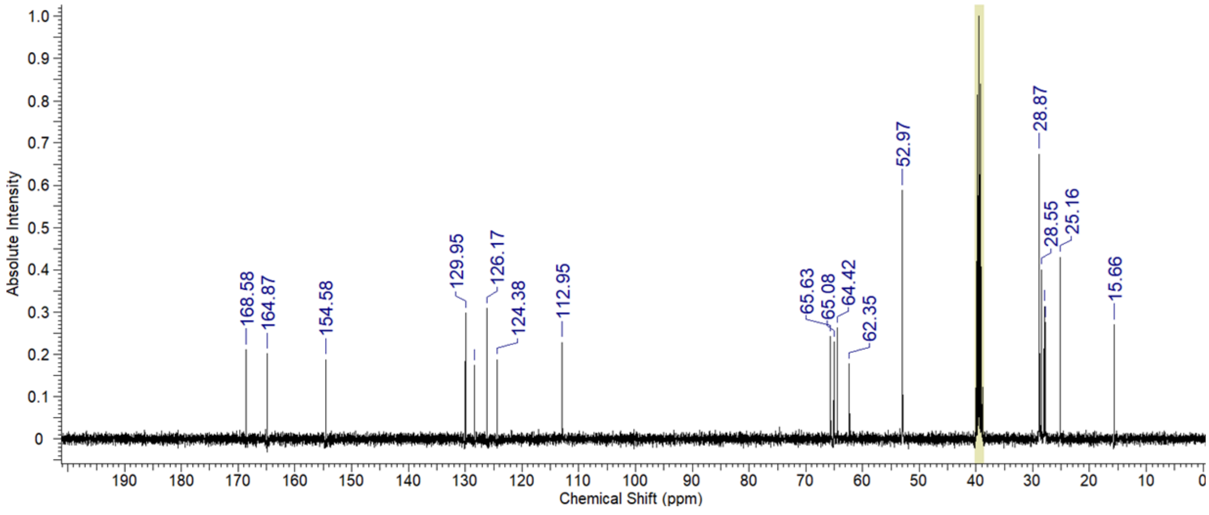

^{13}C NMR (DMSO-d_6) δ [ppm] = 15.7, 25.2, 27.8, 28.0, 28.6, 28.9, 53.0, 62.3, 64.4, 65.1, 65.7, 113.0, 124.4, 126.2, 128.4, 130.0, 154.6, 164.9, 168.6.

Fig. S27. ^{13}C NMR of esterquat **10** ([MCPAC_{12}BET][Br]).

5. FT-IR spectra

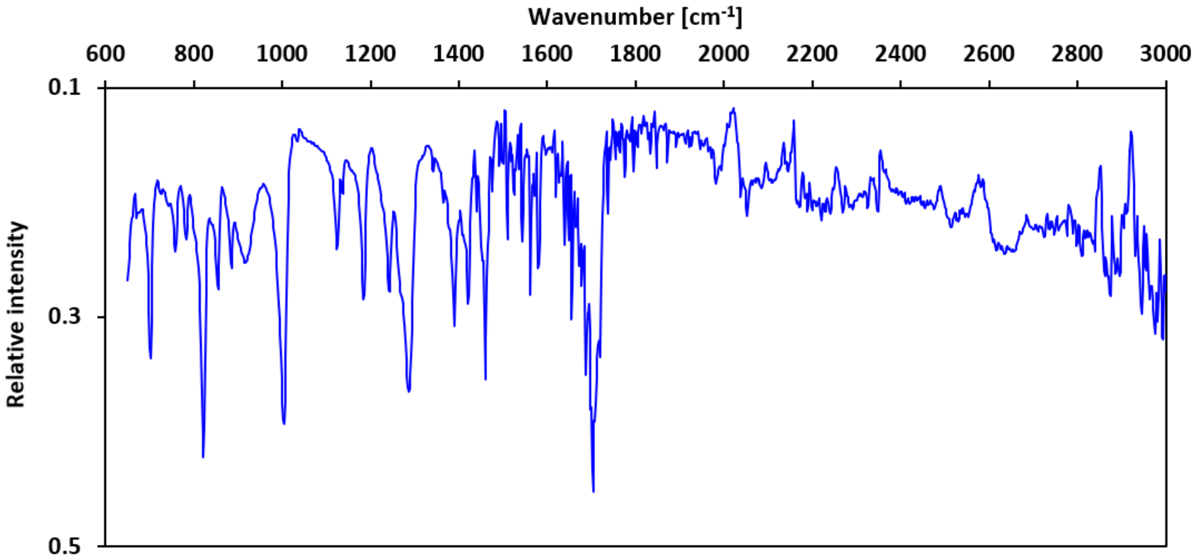

Obtained peaks [cm^{-1}]: 2994, 2975, 1704, 1655, 1562, 1460, 1420, 1391, 1286, 1242, 1184, 1005, 856, 822, 703.

Fig. S28. FTIR spectrum of 2-methyl-4-chlorophenoxyacetic acid (MCPA acid).

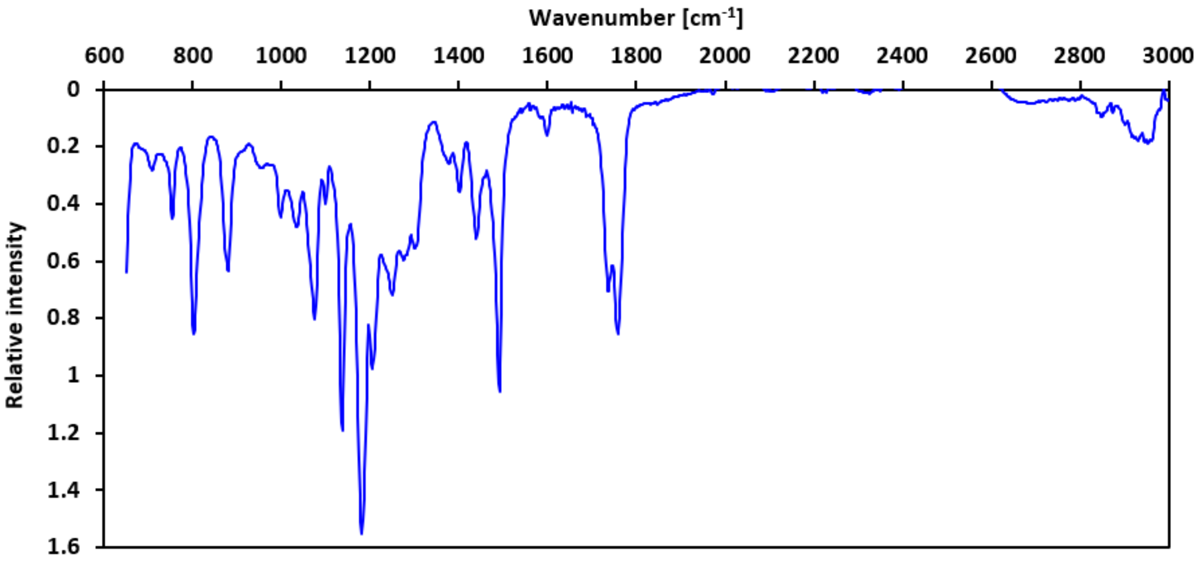

Obtained peaks [cm^{-1}]: 2953, 2930, 1758, 1737, 1491, 1439, 1402, 1249, 1206, 1180, 1135, 1074, 1031, 997, 880, 802, 753.

Fig. S29. FTIR spectrum of ω-bromobutyl 4-methyl-2-chlorophenoxyacetate (**1** - MCPAC_4Br).

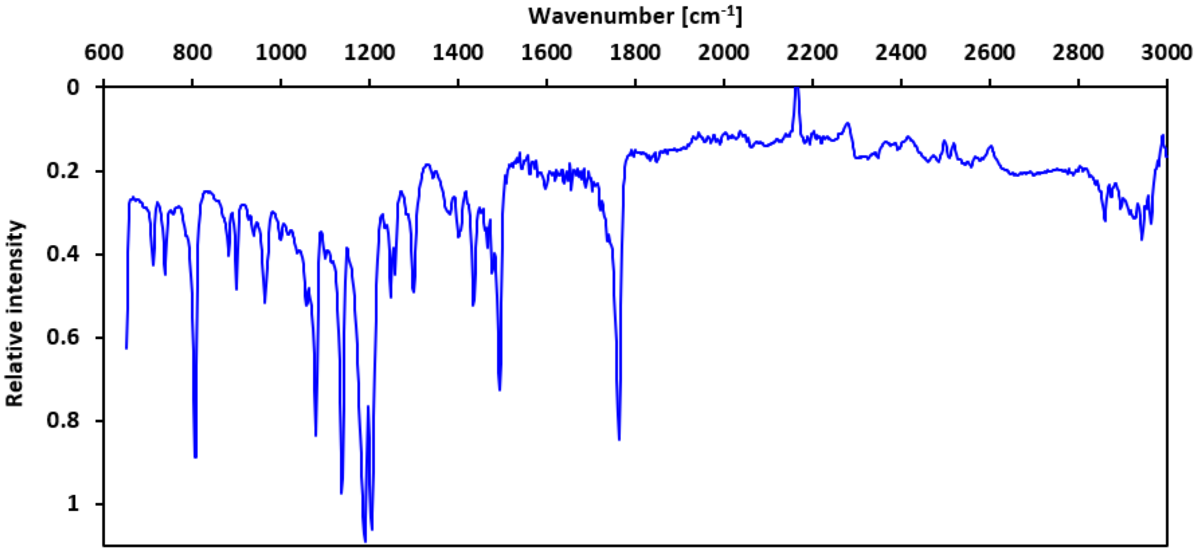

Obtained peaks [cm^{-1}]: 2962, 2943, 2858, 1762, 1493, 1434, 1400, 1299, 1247, 1204, 1189, 1137, 1079, 1057, 964, 899, 882, 807, 738, 710.

Fig. S30. FTIR spectrum of ω-bromohexyl 4-methyl-2-chlorophenoxyacetate (**2** - MCPAC$_6$Br).

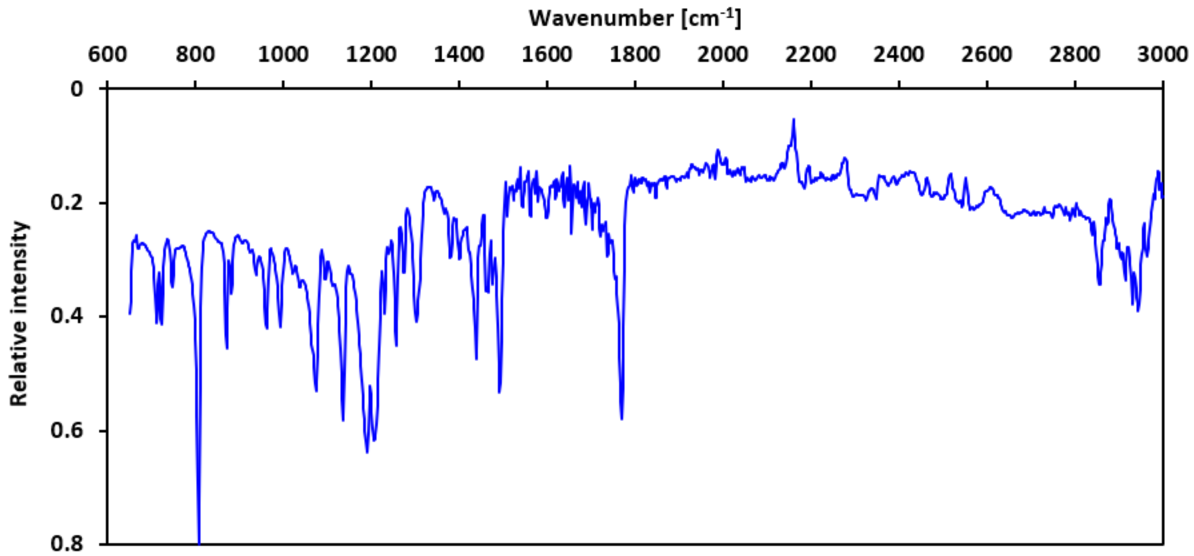

Obtained peaks [cm^{-1}]: 2962, 2943, 2930, 2914, 2854, 1769, 1491, 1461, 1437, 1402, 1381, 101, 1275, 1256, 1206, 1189, 1135, 1074, 992, 962, 871, 807, 748, 723, 712.

Fig. S31. FTIR spectrum of ω-bromooctyl 4-methyl-2-chlorophenoxyacetate (**3** - MCPAC$_8$Br).

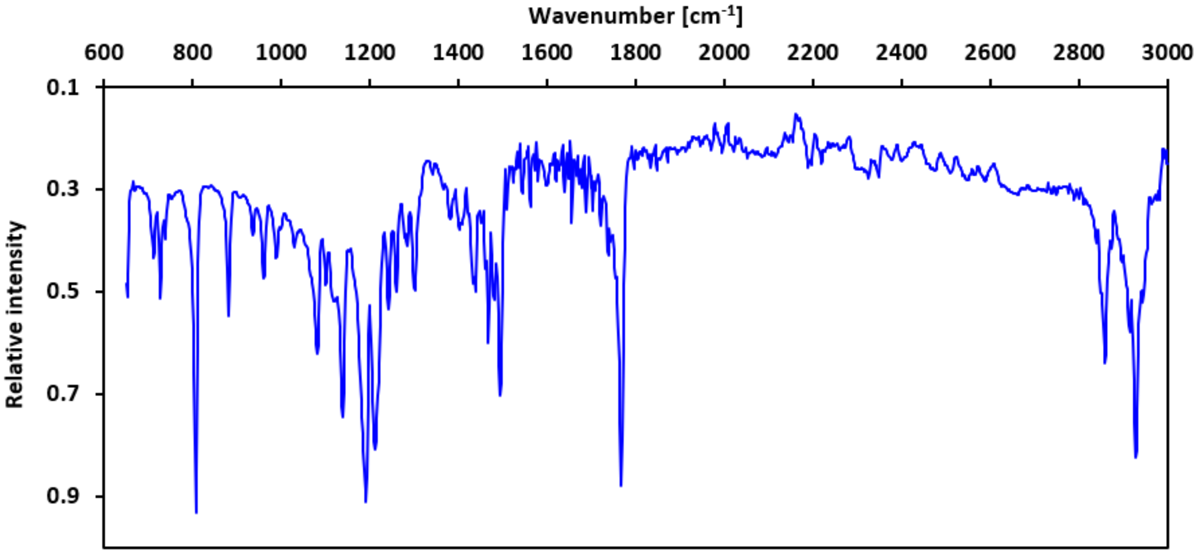

Obtained peaks [cm^{-1}]: 2927, 2858, 1767, 1493, 1467, 1434, 1301, 1260, 1242, 1212, 1191, 1139, 1100, 1081, 1029, 990, 960, 936, 880, 807, 731, 727, 712.

Fig. S32. FTIR spectrum of ω-bromodecyl 4-methyl-2-chlorophenoxyacetate (**4** -MCPAC$_{10}$Br).

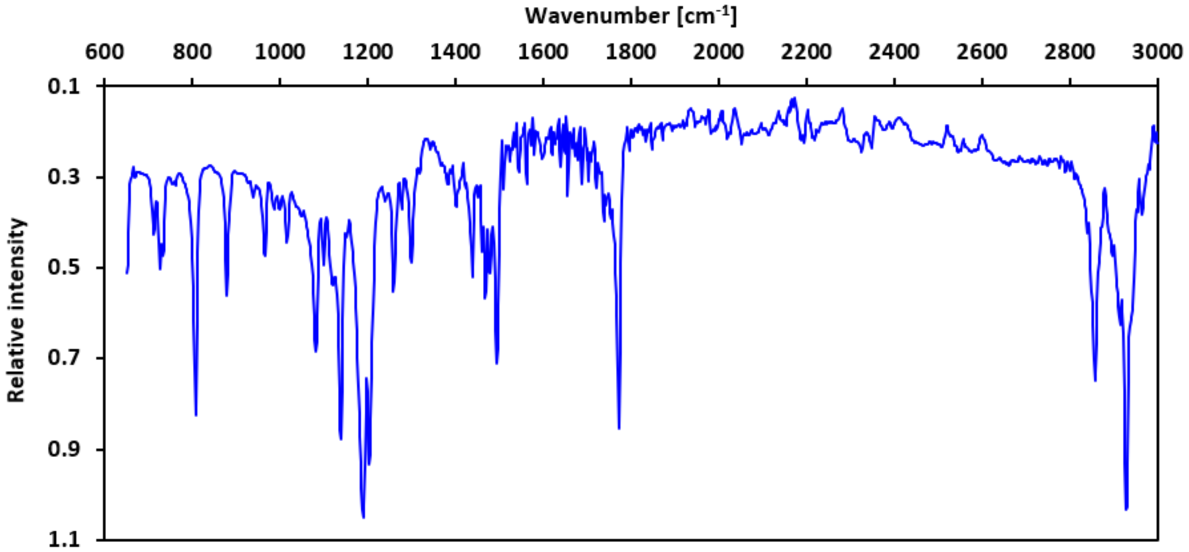

Obtained peaks [cm^{-1}]: 2964, 2927, 2856, 1771, 1655, 1562, 1493, 1467, 1437, 1402, 1299, 1258, 1202, 11891139, 1120, 1098, 1081, 1016, 966, 878, 807, 727, 712.

Fig. S33. FTIR spectrum of ω-bromododecyl 4-methyl-2-chlorophenoxyacetate (**5** -MCPAC$_{12}$Br).

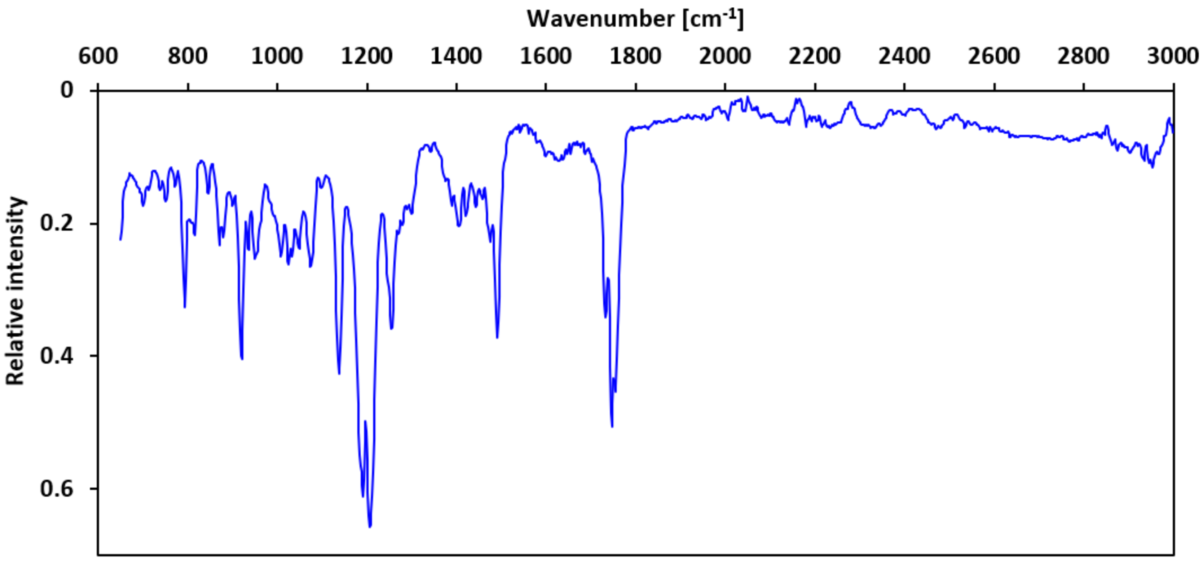

Obtained peaks [cm^{-1}]: 2953, 2934, 1754, 1747, 1732, 1489, 1422, 1409, 1404, 1255, 1206, 1191, 1137, 1074, 1035, 1025, 1008, 951, 921, 871, 846, 716, 794, 751, 701.

Fig. S34. FTIR spectrum of **6** ([MCPAC$_4$BET][Br]).

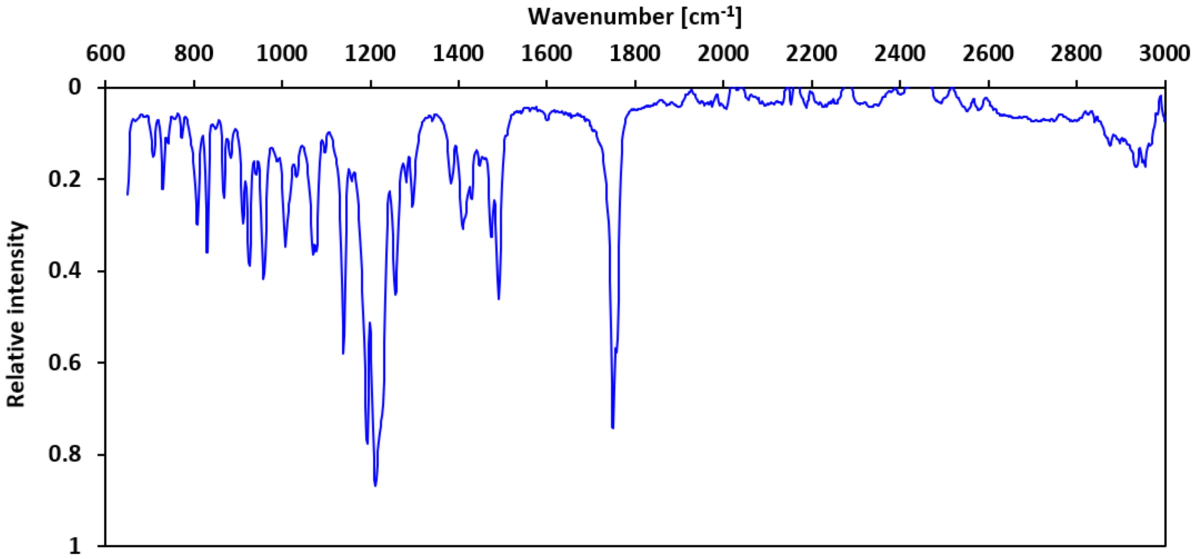

Obtained peaks [cm^{-1}]: 2955, 2930, 1749, 1489, 1473, 1419, 1409, 1381, 1296, 1256, 1212, 1193, 1139, 1070, 1031, 1008, 956, 925, 912, 867, 830, 807, 729, 708.

Fig. S35. FTIR spectrum of **7** ([MCPAC$_6$BET][Br]).

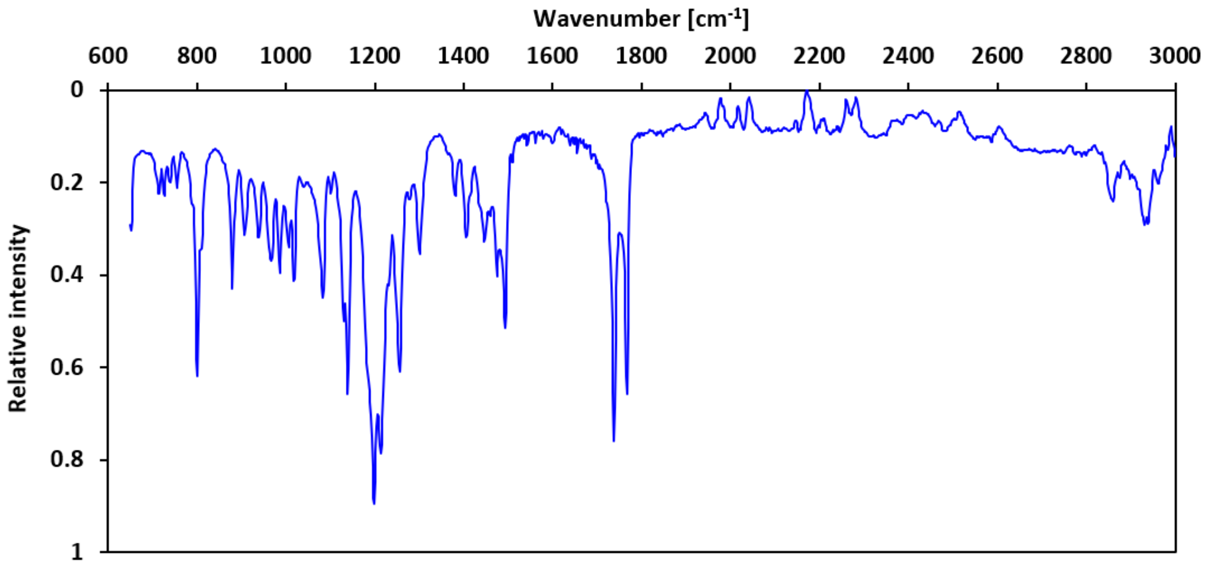

Obtained peaks [cm^{-1}]: 2962, 2938, 2860, 1767, 1737, 1493, 1475, 1447, 1406, 1381, 1301, 1256, 1214, 1199, 1139, 1083, 1018, 1005, 986, 967, 940, 906, 880, 802, 755, 738, 727, 716.

Fig. S36. FTIR spectrum of **8** ([MCPAC$_8$BET][Br]).

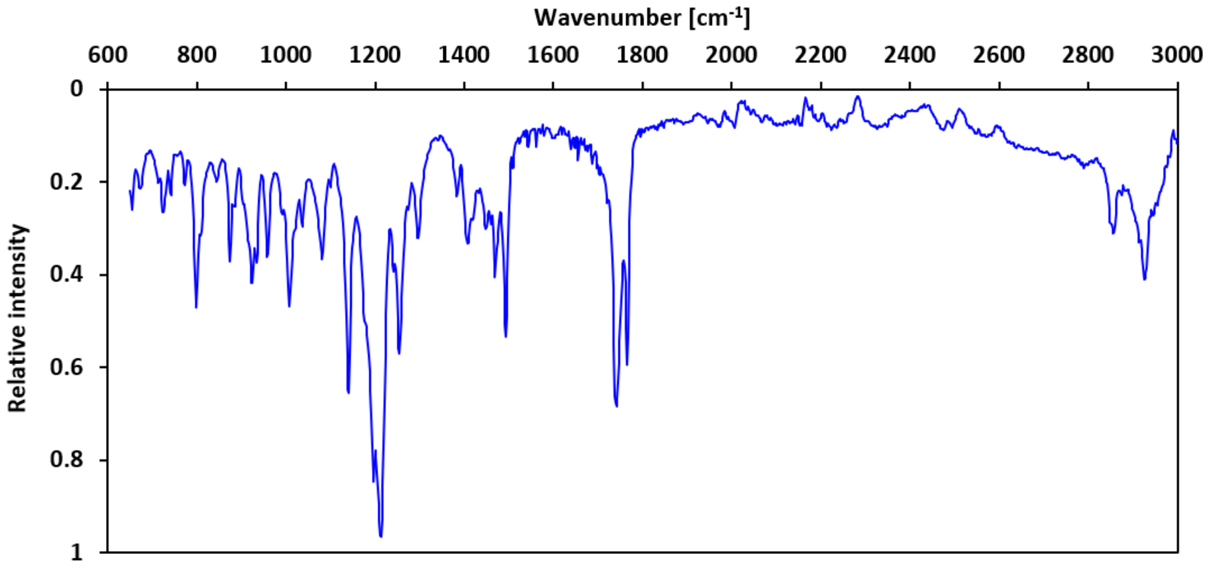

Obtained peaks [cm^{-1}]: 2925, 2856, 1765, 1739, 1493, 1469, 1407, 1381, 1296, 1255, 1212, 1197, 1141, 1081, 1036, 1008, 958, 923, 874, 843, 800, 774, 723, 673.

Fig. S37. FTIR spectrum of **9** ([MCPAC$_{10}$BET][Br]).

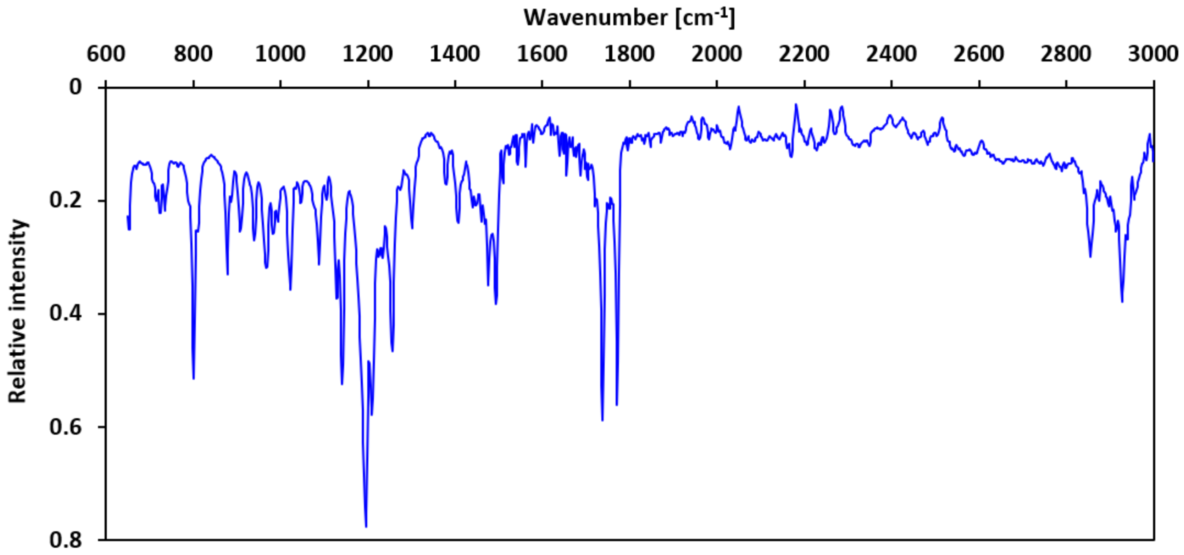

Obtained peaks [cm^{-1}]: 2927, 2854, 1771, 1737, 1493, 1476, 1407, 1378, 1301, 1255, 1210, 1195, 1141, 1128, 1089, 1022, 966, 940, 908, 878, 802, 734, 723.

Fig. S38. FTIR spectrum of **10** ([MCPAC$_{12}$BET][Br]).

6. MS Spectra

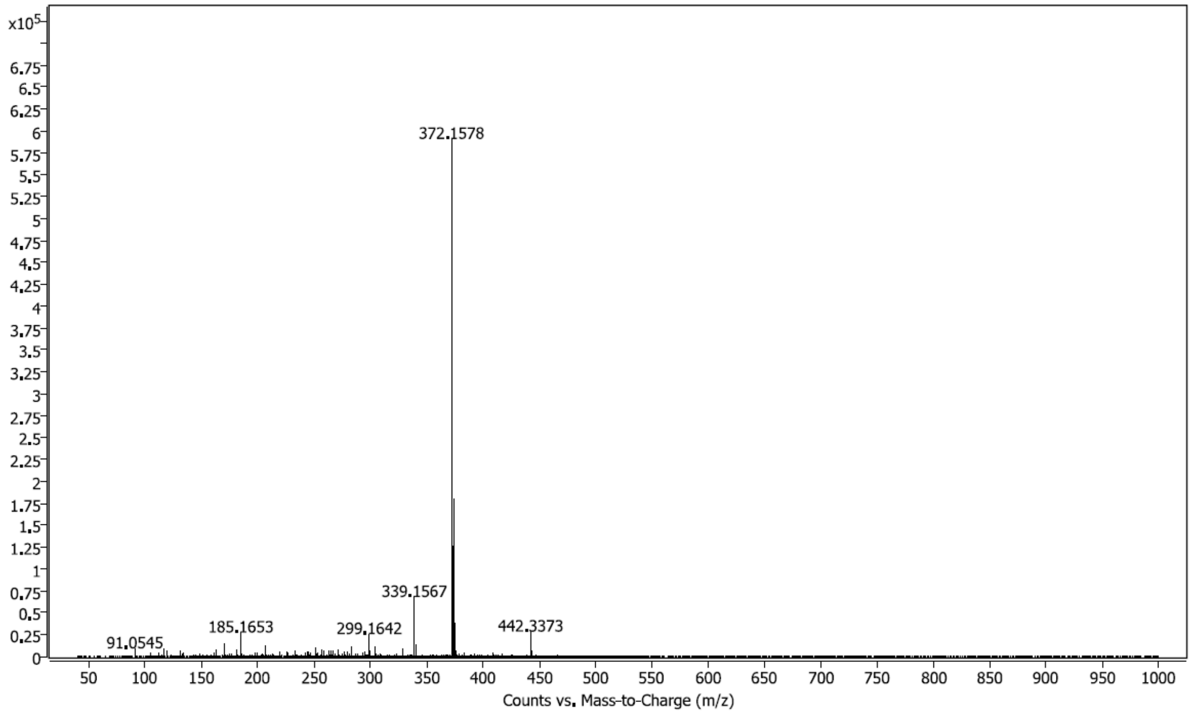

Fig. S39. MS spectrum of esterquat **6** ([MCPAC$_4$BET][Br]).

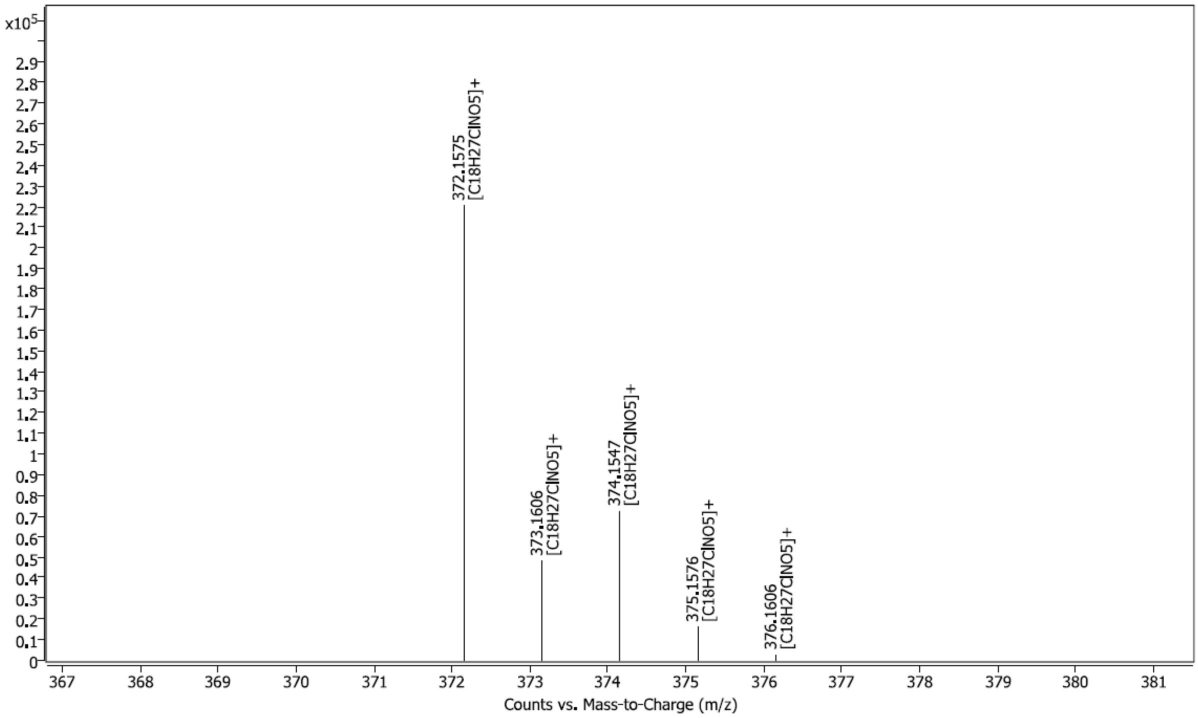

Fig. S40. Enlarged fragment of MS spectrum of esterquat **6** ([MCPAC$_4$BET][Br]).

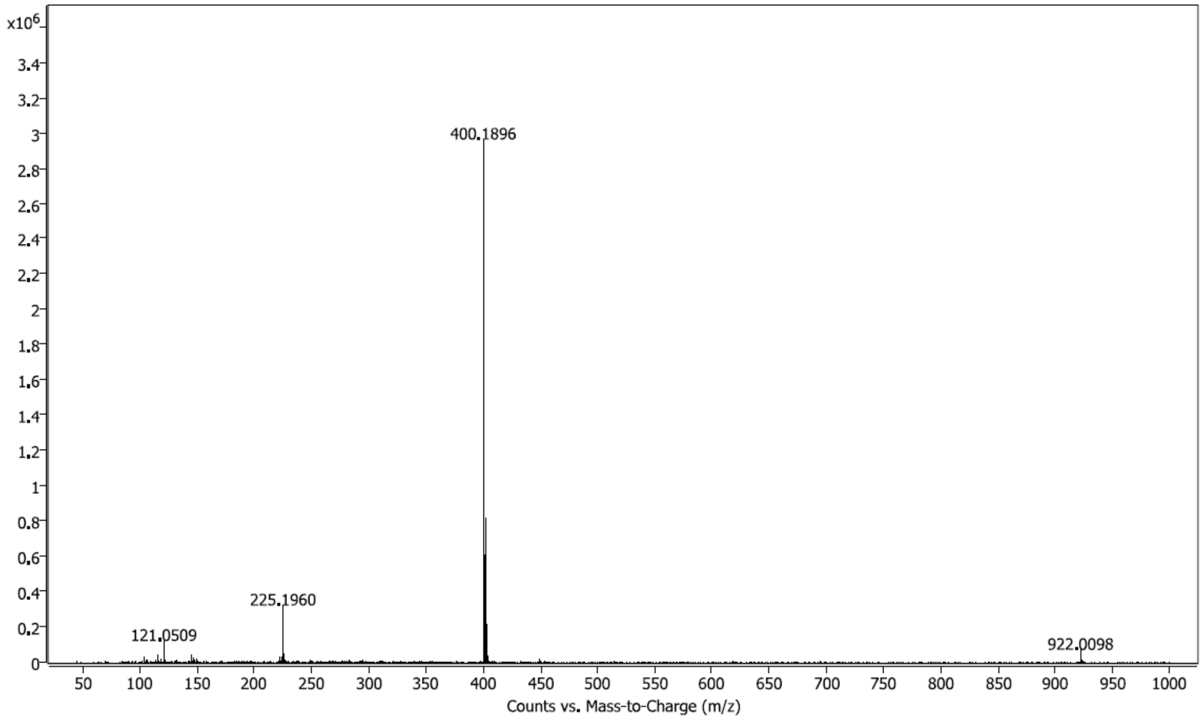

Fig. S41. MS spectrum of **7** ([MCPAC$_6$BET][Br]).

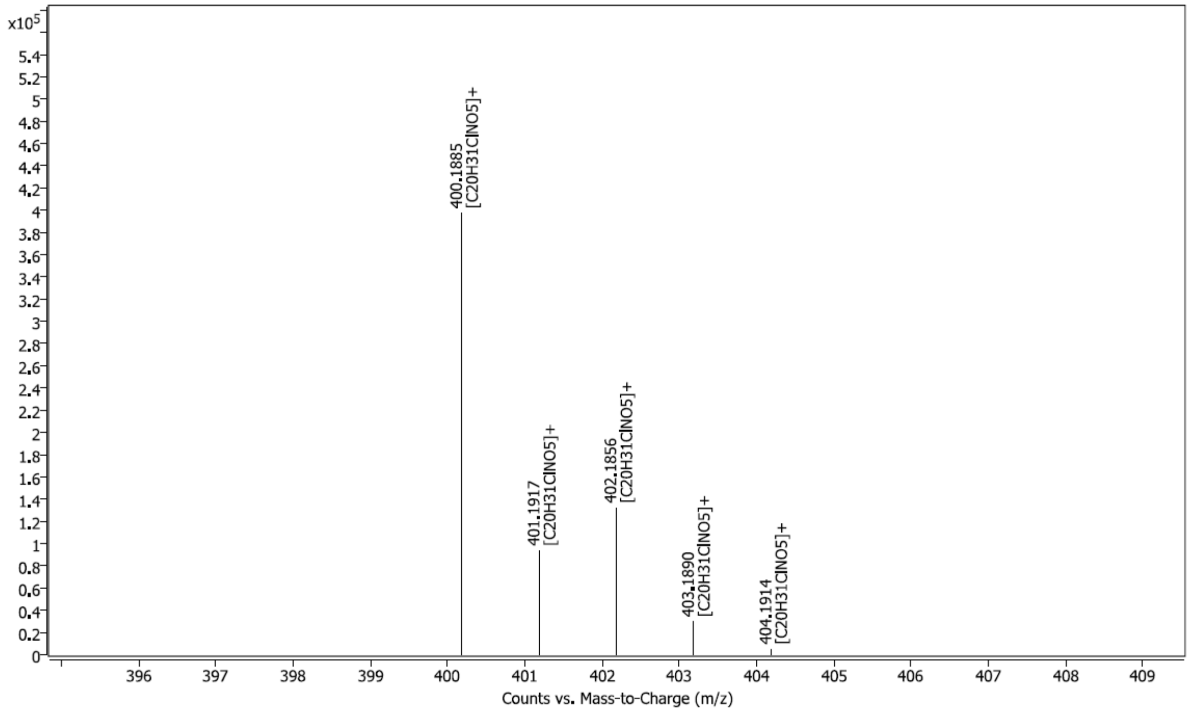

Fig. S42. Enlarged fragment of MS spectrum of **7** ([MCPAC$_6$BET][Br]).

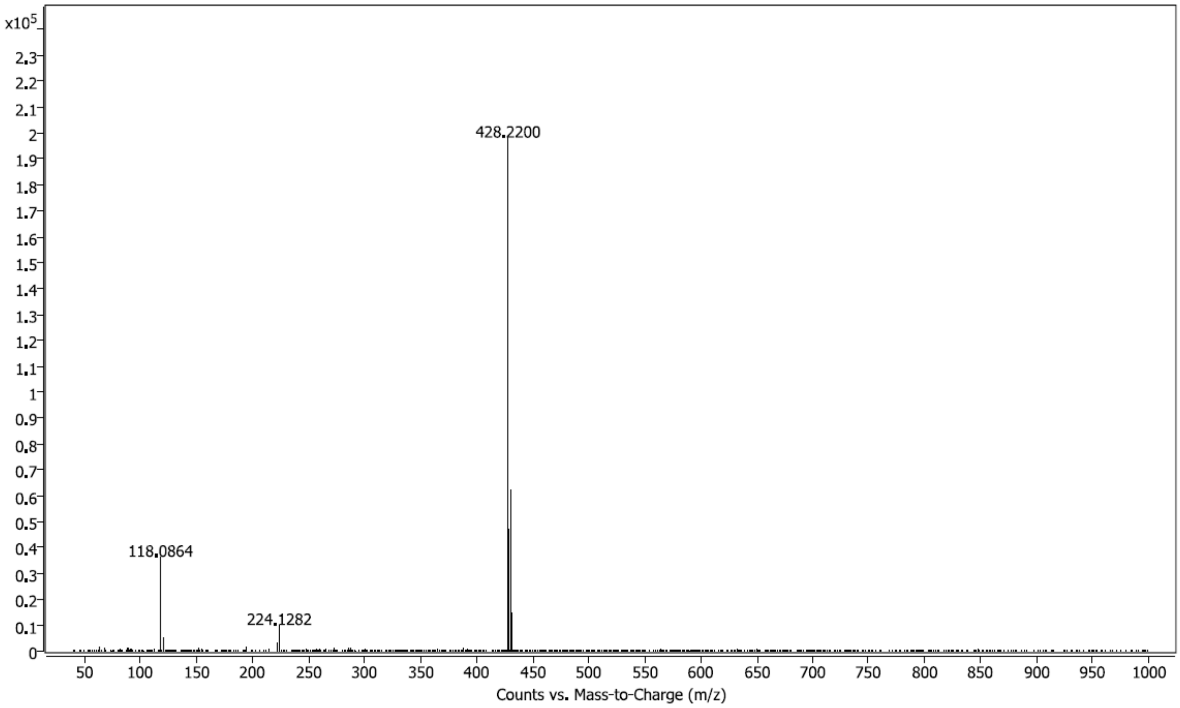

Fig. S43. MS spectrum of **8** ([MCPAC$_8$BET][Br]).

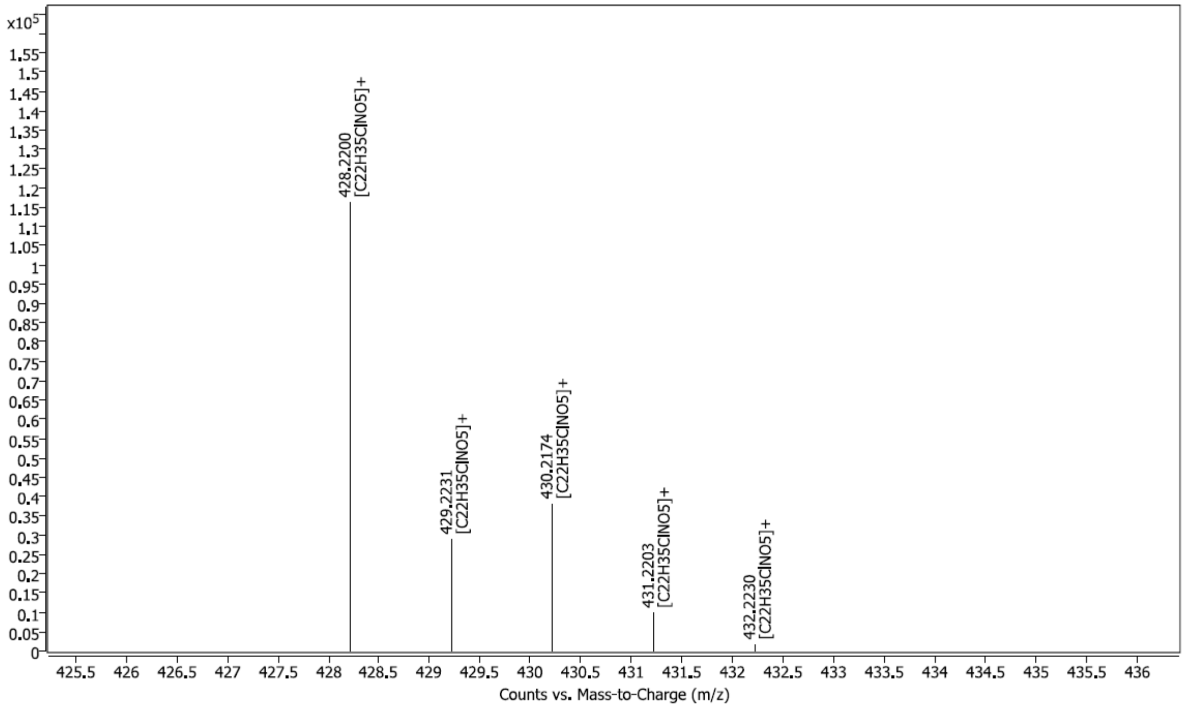

Fig. S44. Enlarged fragment of MS spectrum of **8** ([MCPAC$_8$BET][Br]).

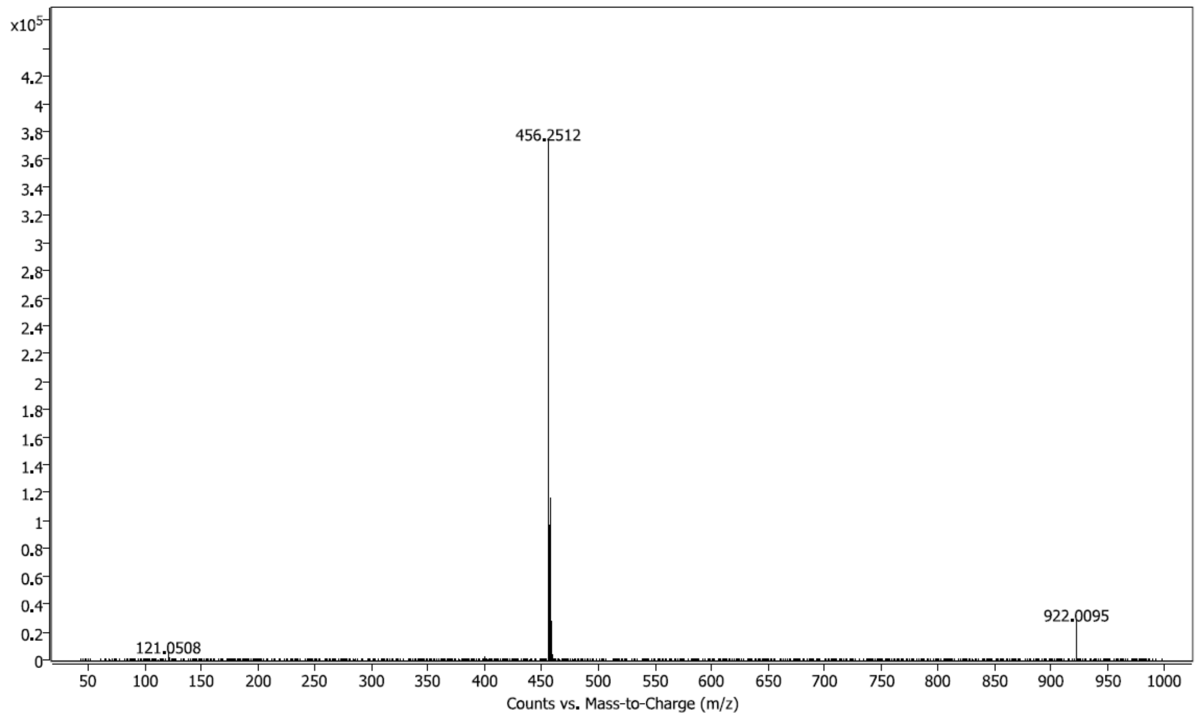

Fig. S45. MS spectrum of **9** ([MCPAC$_{10}$BET][Br]).

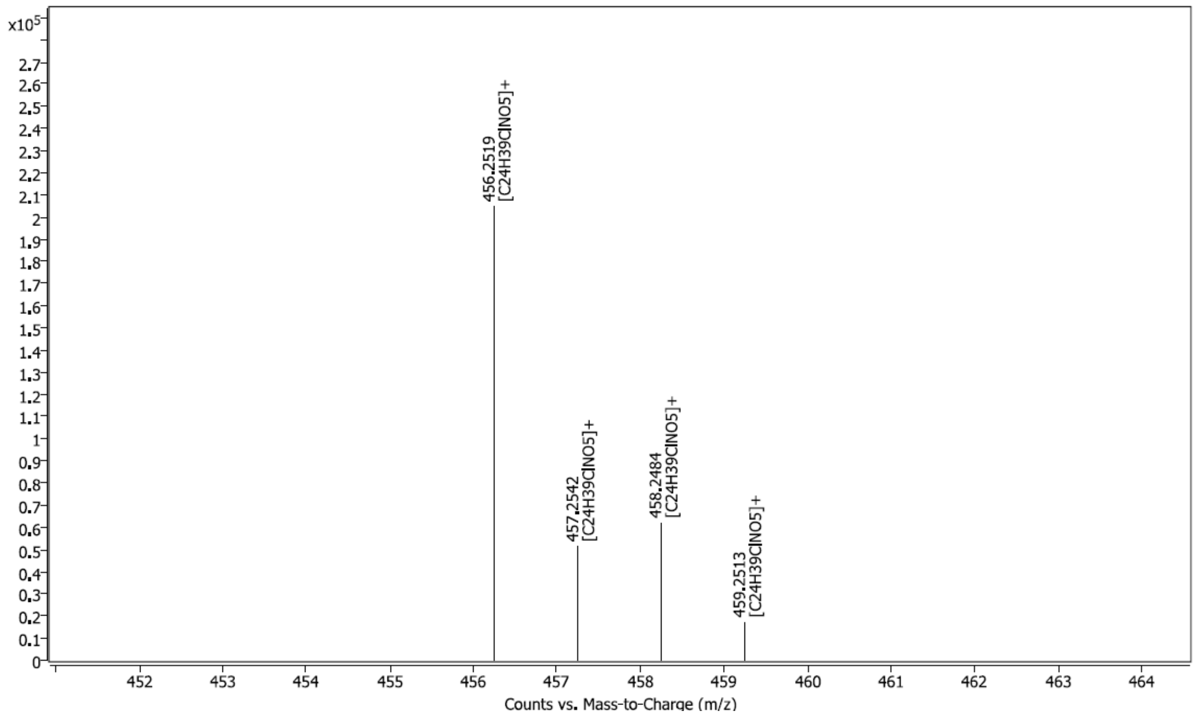

Fig. S46. Enlarged fragment of MS spectrum of **9** ([MCPAC$_{10}$BET][Br]).

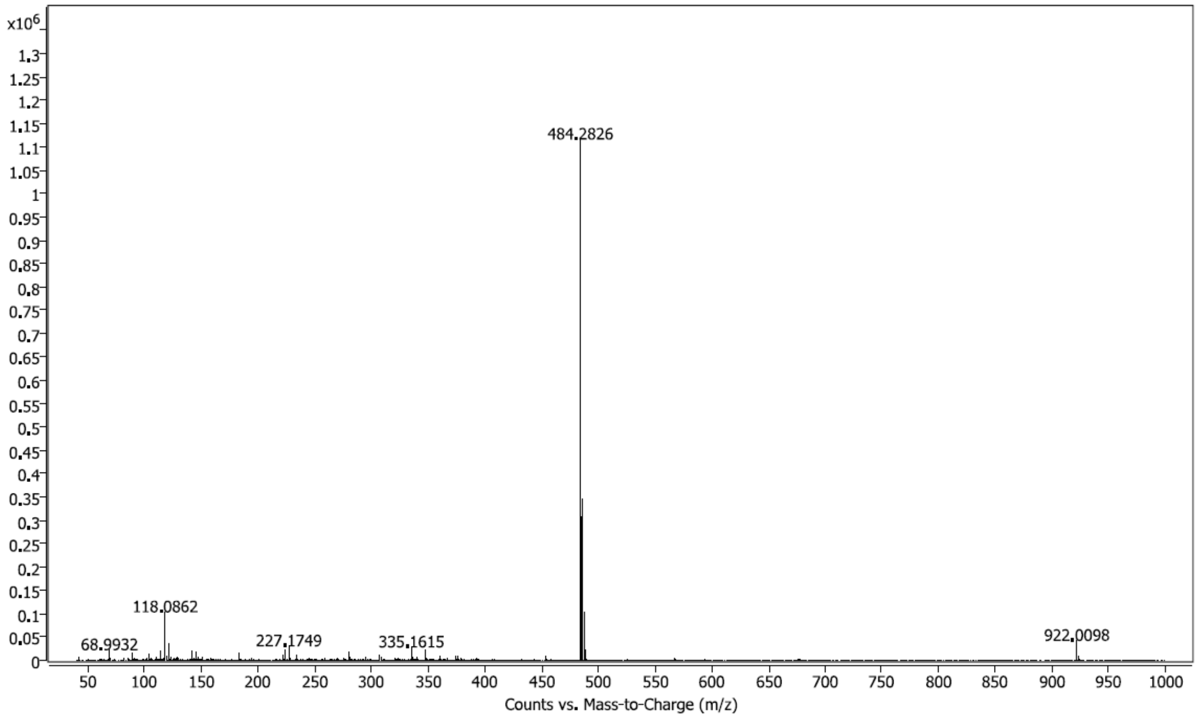

Fig. S47. MS spectrum of **10** ([MCPAC$_{12}$BET][Br]).

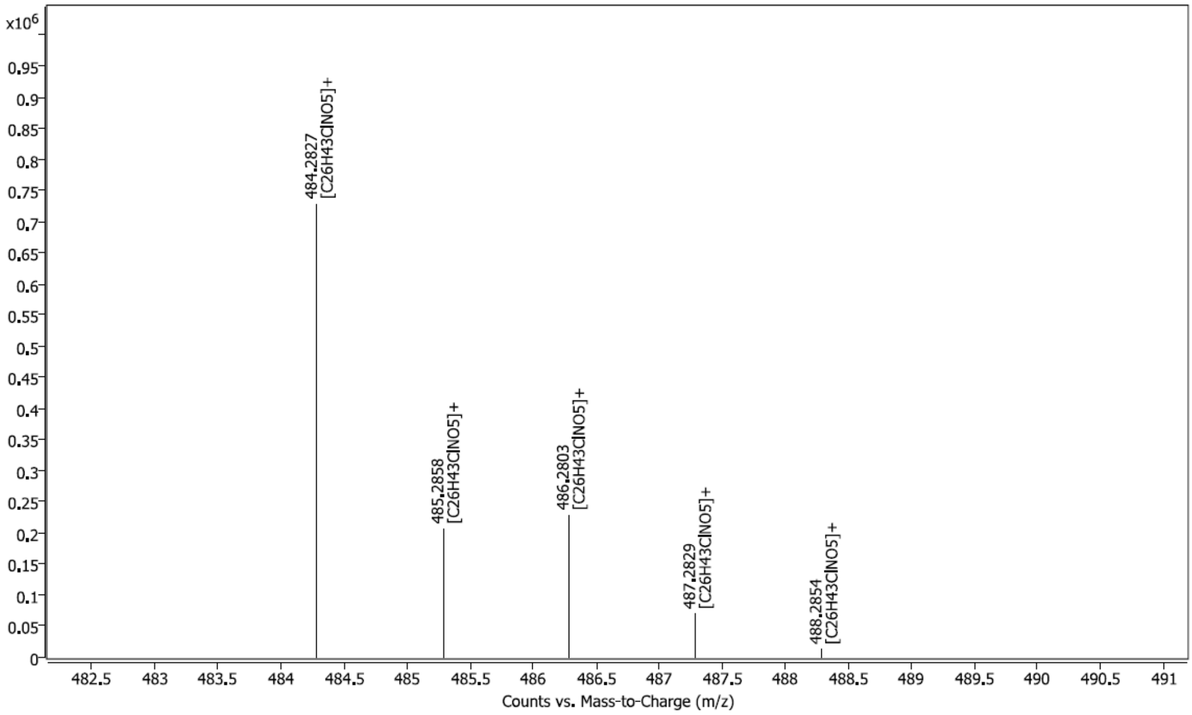

Fig. S48. Enlarged fragment of MS spectrum of **10** ([MCPAC$_{12}$BET][Br]).

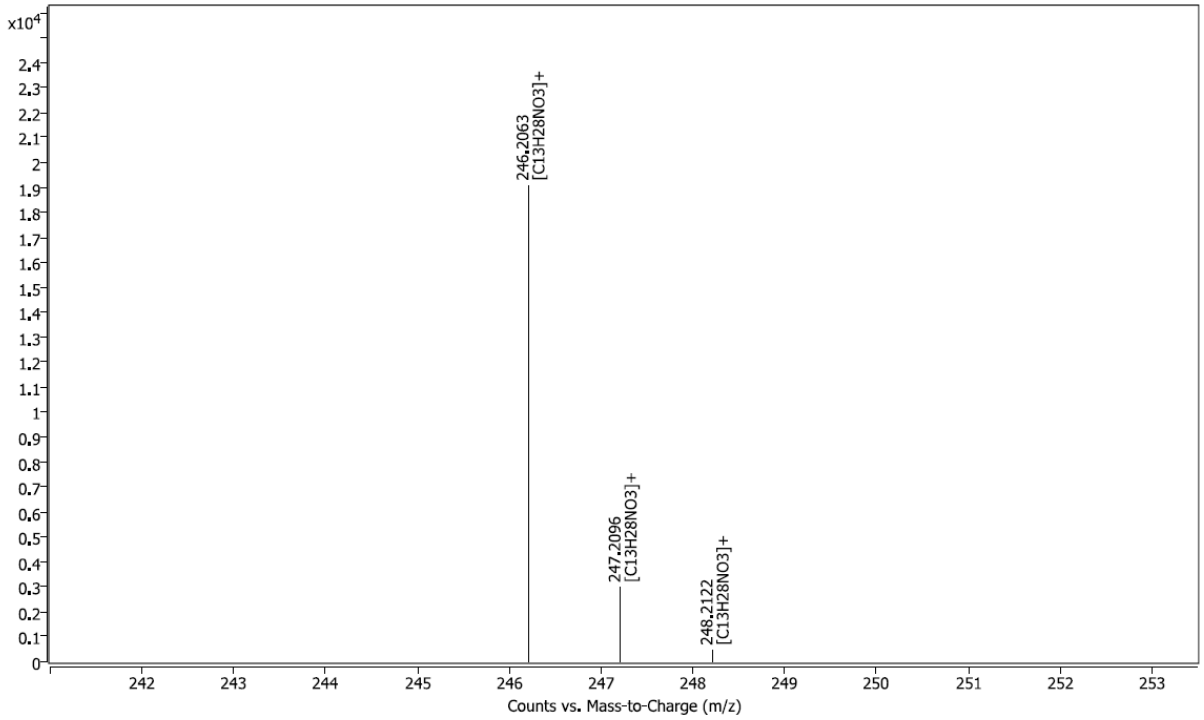

Fig. S49. MS spectrum of ion used to assess concentration of primary biodegradation product in hydrolysis (BETC$_8$OH).

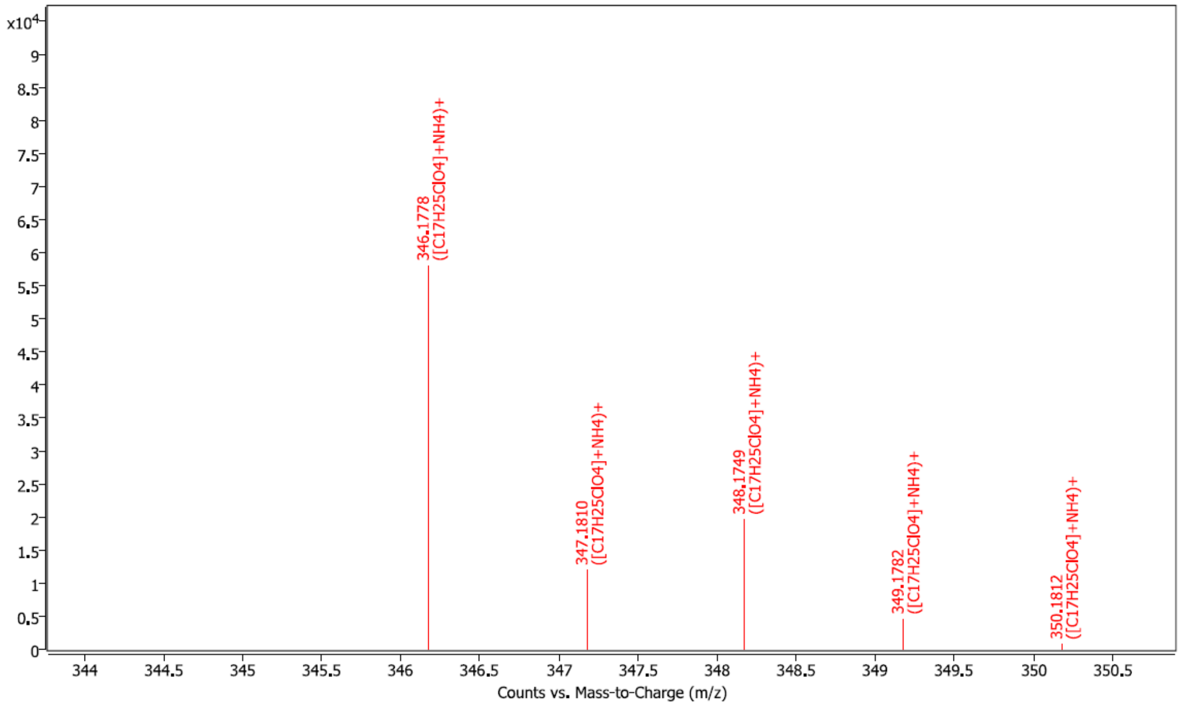

Fig. S50. MS spectrum of ion used to assess concentration of primary biodegradation product in hydrolysis (MCPAC$_8$OH).

7. Phytotoxicity towards plants

Phytotoxicity of the obtained compounds toward plants was tested on white mustard (*Sinapis alba* L.). The seeds were sown in plastic pots with a diameter of 12 cm filled with commercial soil. Plants were grown in 18 h day/6 h night photoperiod in 25 °C by day and 22 °C by night and 40–60% humidity all day for two weeks. Afterwards, the plants were thinned to five per pot. The greenhouse experiment was designed as a randomized experiment with 5 replicants. When plants were at four-leaf stage, aqueous solutions of the examined substances were applied on them by using hand sprayer. Esterquats (**6-10**) and reference commercial product (chwastox extra 300 SL) Esterquats **6-10** were dissolved at the concentration of 10 mmol dm^{-3}, which corresponds to 400 g of active substance (MCPA) per 200 dm^3 of spray solution per 1 ha. In effect, it was ensured that the same amount of ions of each salt were in a direct contact with tested plants. Test included a control group of plants treated with deionized water alone. In order to ensure the proper pressure in the sprayer and the volume of the sprayed solution containing the test compound, five preliminary sprays (separately for each solution) were performed, which were collected in a receiving vessel.

Solutions of tested substances were applied when the plants were at the four to six leaf stage (BBCH 14-16) After treatment, the plants were again placed in a greenhouse under the environmental conditions mentioned above. Fresh weight of plants was measured two weeks after treatment (Fig. S51) using a technical balance with 0.01 g accuracy (ME2002, Mettler Toledo, Switzerland). To properly assess significant differences in the determined phytotoxic effects, a statistical analysis was performed for all biological experiments. The collected data were analyzed using Microsoft Excel 2016 Analysis ToolPak. First, the one-way analysis of variance (ANOVA) test was performed to reveal if there were statistically significant differences between the tested groups (p value < 0.05 means that there were statistically significant differences in the studied groups). Tukey's post-hoc test was used to calculate HSD (honestly significant difference) at the 5% level of significance. Different lowercase letters indicate significant differences between treatments. Results were presented in Table S4.

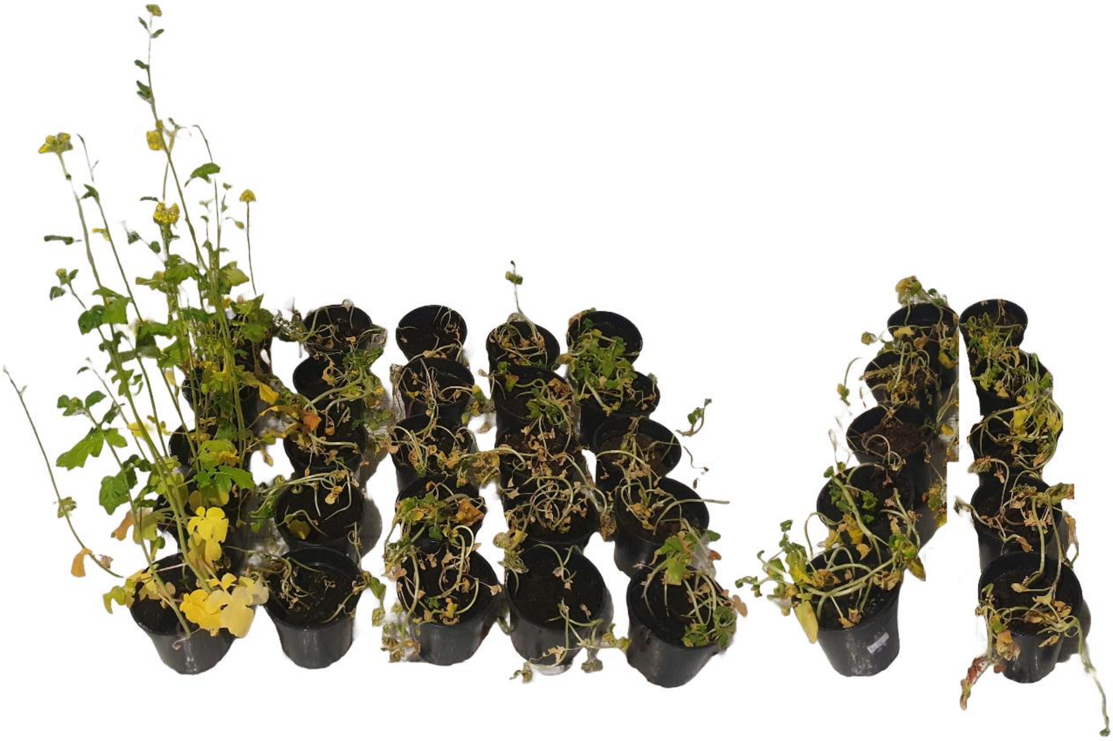

Fig. S51. White mustard plants treated with synthesized products 2 weeks after treatment. From left: distilled water (control), esterquats **6-10** and chwastox extra 300 SL (reference)**.**

Table S4. Herbicidal activity of analyzed compounds.

Compound	n[a]	Mass reduction (%)	SE[b] (%)	Statistical analysis (Group)
**6**	4	58	8	a
**7**	6	59	4	a
**8**	8	58	2	a
**9**	10	53	7	a
**10**	12	47	9	a
**Chwastox extra 300 SL**		48	6	a

[a] Number of carbon atoms in the alkyl spacer, [b] SE – standard error
